# Cyclophane-Based Dendrimers: Today and Tomorrow

**DOI:** 10.3390/molecules30214211

**Published:** 2025-10-28

**Authors:** Olga Mostovaya, Alena Vavilova, Asiya Gazizova, Ivan Stoikov

**Affiliations:** A.M. Butlerov Chemical Institute, Kazan Federal University, Kremlevskaya St., 18, 420008 Kazan, Russia; olga.mostovaya@mail.ru (O.M.); anelia_86@mail.ru (A.V.); asiya_gazizova@mail.ru (A.G.)

**Keywords:** dendrimers, pillararenes, resorcinarenes, (thia)calixarenes, synthesis, application, drug delivery

## Abstract

For decades, dendrimers have attracted the great interest of researchers, and continue to do so to this day. These compounds found a wide variety of applications in such areas as medicine, catalysis, and electronics. However, their synthesis is very expensive, and purification and isolation are difficult. In addition, dendrimers are often highly toxic. Toxic properties are significantly dependent on the generation and terminal group features. For the toxicity, decreasing the modification of dendrimer structure is widely used. This review discusses some examples of constructing dendrimers based on macrocyclic compounds—cyclophanes (pillararenes, resorcinarenes, and (thia)calixarenes). Preparation of these hybrids is not very difficult, and due to the presence of a hydrophobic macrocyclic platform, they acquire a number of practically useful properties that are not available to traditional dendrimers, such as additional opportunities for encapsulating substrates. Moreover, dendrimers acquire amphiphilic and chiral properties. This review is devoted to the features of the synthesis and properties of macrocyclic dendrimers based on cyclophanes. The review also demonstrates the prospects of using the resulting dendrimers for medicine, sensorics, catalysis and alternative energy sources.

## 1. Introduction

The history of dendrimer chemistry goes back more than forty years. It began in 1978 when F. Vögtle and colleagues published the description of the synthesis of “cascade molecules” [1]. Dendrimer chemistry was significantly advanced with the works of D. A. Tomalia [2,3], who was also the author of the “dendrimer” term itself. Dendrimers are nanosized radially symmetrical synthetic macromolecules with definite homogeneous and monodisperse structure. These compounds have symmetrical branched units, usually named “branches”, which are placed around the cylindric or linear core, so having a structure similar to a tree [4,5,6]. The word “dendrimers” originates from the Greek words “dendron”, which means tree-like, and “meros”, which means part or unit. During all these decades, a great scientific interest in this class of polymer compounds constantly remained. To date, a voluminous data set about the synthesis and application of a wide variety of dendrimers has been accumulated. This resulted in a huge number of literature reviews describing both the synthesis and application aspects of dendrimer chemistry due to the wide range of the attractive properties of these compounds. Considering that these compounds are analogous to polymers, the dendrimers can go beyond them due to their strictly defined and symmetrical structure and monodispersity. Such properties are provided by an important synthetic feature of this type of polymer: the growth of the molecule occurs as a result of completely controlled processes [7]. In addition to dendrimers widely used for catalysis, they can also be proposed as drug delivery agents, contrast agents for magnetic resonance imaging (MRI), transfection agents, etc. [8,9]. Such a diverse application of these hyperbranched compounds is due to their wide possibilities of binding a wide variety of compounds. This binding can occur both in the internal cavities of dendrimers (encapsulation) and via terminal functional groups. Covalent binding with prodrugs can also be used, along with the formation of entire ensembles of dendrimers and guest molecules [10]. Having a large number of terminal and internal functional groups, dendrimers have the ability to take part in multifunctional interactions. It allows them to bind the multifunctional biomolecules, such as proteins and DNA with the following so-called dendriplexes formation, which is useful for biopolymer delivery into living cells [11,12]. Another important property of dendrimers, namely their high solubility in water, determines their attractiveness for medical applications [13]. In the presence of a wide range of binding groups, such solubility can dramatically facilitate the hydrophobic drugs used in biological fluids. Currently, a number of dendrimers have already been tested clinically, and now they are patented as drug delivery agents [14]. Thus, the Australian company Starpharma has a series of patents for using of the dendrimers in pharmaceuticals [15]. At the same time, these compounds have some disadvantages limiting their practical application. So, usually dendrimers have high cytotoxicity, and their production is too expensive, especially those ones with high generations [16]. However, the functionalization of dendrimers often aimed to reduce their insufficient properties’ effect [17].

**Figure 1:**
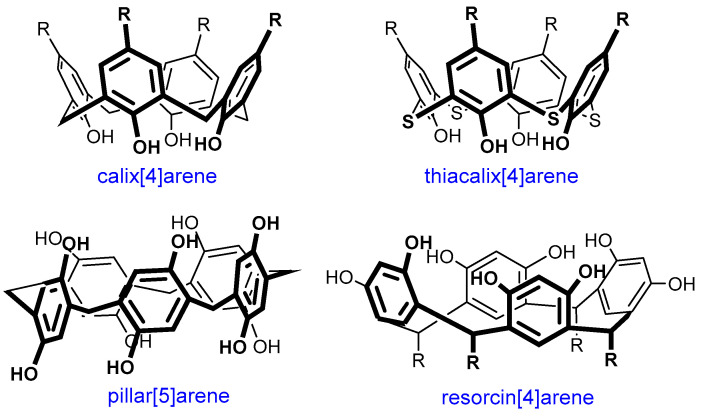
The structures of cyclophane macrocycles.

A promising direction for dendrimer modification is their combination with macrocyclic compounds [18]. Different macrocycles have found wide applications in medicine. Today, there is a large number of drugs based on them and now these compounds are studied extensively [19]. There are antitumor and antimicrobial drugs, immunosuppressors, as well as drugs for the treatment of autoimmune disorders among them [20]. The high demand for macrocycles in medicine is due to their attractive properties. Due to their structure, these compounds can strictly fix the binding centers, which allows them to enhance the selectivity of their binding to targets with complex geometry (large flat, groove-shaped, or tunnel-shaped), which cannot be accessed by conventional non-cyclic molecules. As a rule, natural macrocycles, namely oligopeptides, are produced by living organisms, and can be used as drugs. Synthetic macrocycles are usually obtained by natural analog modifying, which results in their properties’ improvement [21,22]. Carbohydrate compounds, in particular, cyclodextrins, are widely used. For example, the drug Sugammadex (tradename Bridion^®^) is used in surgery to relieve neuromuscular blockade [23]. Currently, medicinal chemists are still trying to obtain the analogs of other macrocycles, such as pillar[n]arenes, calix[n]arenes, terphen[n]arene, which are capable of binding the neuromuscular blocking agent [24,25,26,27]. In general, the growing interest in macrocycles, as well as in dendrimers, has not waned for many decades since the time of their discovery [28,29]. In addition to their application in medicine, they can be used in different areas: food industry, nanotechnology, catalysis, and also as chemical sensors, as selective extraction agents, etc. [30,31,32,33,34,35,36,37,38]. As a result of the long-term investigation of different types of macrocyclic compounds, the efficient approaches to their functionalization have been developed already. The simplicity of macrocycles’ modification and the presence of a number of functional groups make the prospects for their applications obvious—for example, as the core of dendrimers, as well as terminal groups or branching nodes. Moreover, using macrocyclic compounds in the design of dendrimers gives additional possibilities for binding various substrates. As a result, alongside the terminal groups and internal cavities of dendrimers, the cavity of the macrocycle itself can also be involved in complex formation. One of the most numerous groups of promising macrocyclic compounds are cyclophanes, such as (thia)calixarenes, resorcinarenes and pillararenes [39,40,41,42] (Figure 1). Their popularity among researchers is provided by several reasons simultaneously. The conformational diversity of (thia)calixarenes and resorcinarenes makes it easy to obtain a wide variety of compounds with the groups which are spatially located in a strictly defined way due to the macrocycle lower rim functionalization. This is the reason for multivalent effect occurrence [43]. The cyclophane platforms themselves are hydrophobic, so the ease of their functionalization makes it possible to obtain a set of compounds with any hydrophilic–hydrophobic balance required.

Dendrimers with macrocyclic (cyclophane) components, no matter if they are the cores or the fragments of branches, have another fundamental difference from the conventional ones. They can be classified as biomimetic systems. These dendrimers demonstrate such properties due to their unique structure. For example, pillararenes are well-known for their planar chirality [44]. Although the production of such compounds is rather complicated due to the rotation of phenylene groups around the bridging methylene fragments, there are many approaches to the production and separation of chiral pillararenes developed [45]. Resorcinarenes obtained within the condensation reaction of resorcinol with aliphatic and aromatic aldehydes, also have chiral properties in some conformations [41]. It is known that chiral molecules (amino acids, sugars, as well as proteins, nucleic acids, hormones and enzymes based on them) are the vital compounds on Earth [46,47]. Thus, if conventional dendrimers require the functionalization with appropriate groups to impart chiral properties, the cyclophane macrocycles have chiral properties due to geometric characteristics of the platform already. All of this provides their ability of selective binding to chiral biomolecules. Such a feature opens up excellent prospects for using macrocyclic dendrimers as catalysts for both asymmetric synthesis and medical technologies.

In this review, the dendrimers containing macrocyclic cyclophane fragments as cores or as surface functions are discussed. The examples of cyclophane dendrimers formed by supramolecular interactions between macrocyclic fragments and other groups (similar to tectodendrimers) are also given [48]. Special attention is paid to the prospects of the application of these hybrid molecules for various branches of industry and science.

## 2. Dendrimers with Cyclophane Fragments as a Core

### 2.1. Dendrimers Based on Pillararenes

**Scheme 1:**
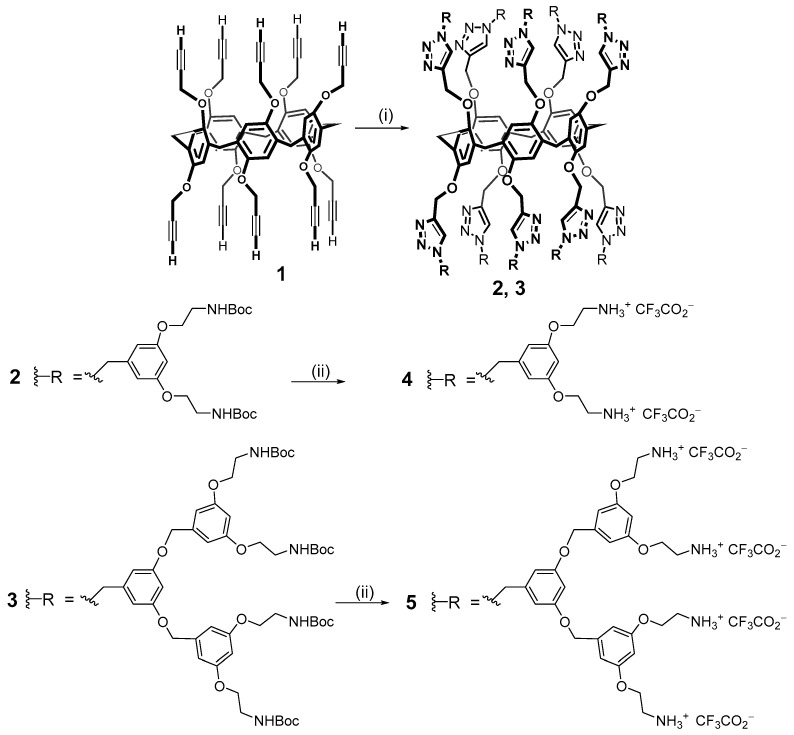
Reagents and conditions: (i) R-N_3_, CuSO_4_**·**5H_2_O, sodium ascorbate, CH_2_Cl_2_/H_2_O (**2**: 88%, **3**: 81%); (ii) TFA (**4**: quantitative, **5**: quantitative).

As noted above, macrocyclic fragments can be promising as dendrimer cores. Macrocycles with functionalized groups are excellent templates to obtain branched molecules providing a shape which is close to spherical. In addition, macrocyclic compounds have the cavity for guest binding, which provides products with the properties of selective complexing agents. The most convenient way to investigate macrocyclic dendrimers is to become acquainted with the youngest and still a small class of compounds named pillararenes. This class appeared much later than the (thia)calixarenes and resorcinarenes described above [42,49] but it attracts the attention of many researchers nowadays. These macrocycles have great potential as complexing agents, while a large number of functionalized hydroxyl groups (ten) allow us to obtain products with good solubility in aqueous medium [50,51]. An important feature of pillararenes is their planar chirality which demonstrates good water solubility along with broad prospects for the application in biomedical technologies. Currently, only a few examples of dendrimers based on this class of cyclophanes are presented in the literature but they have already satisfied expectations. Thus, the first representatives of dendrimers based on pillar[5]arene **2**–**5** were obtained in 2013 by the convergent method upon the copper-catalyzed alkyne–azide cycloaddition (CuAAC) reaction from **1** (Scheme 1).

Derivatives of the first and second generations **2** and **3** with dendrons based on peripheral benzyloxycarbonyl (Boc)-protected amine sub-units on both the rims of the macrocycle were obtained in high yields (81 and 88%, respectively) [52]. Then, the protective groups were quantitatively removed with a large excess of trifluoroacetic acid. The resulting dendrimers **4** and **5** were capable of binding plasmid DNA (pCMVLuc) with the following stable positively charged particle formation. These polyplexes were evaluated in 5% glucose or 150 mM NaCl aqueous solutions and tested in vitro on HeLa cells. It turned out that in glucose solutions, they had the optimal sizes for cellular uptake (up to 60 nm). At the same time, the particle sizes in 150 mM NaCl were significantly larger (up to 930 nm).

**Scheme 2:**
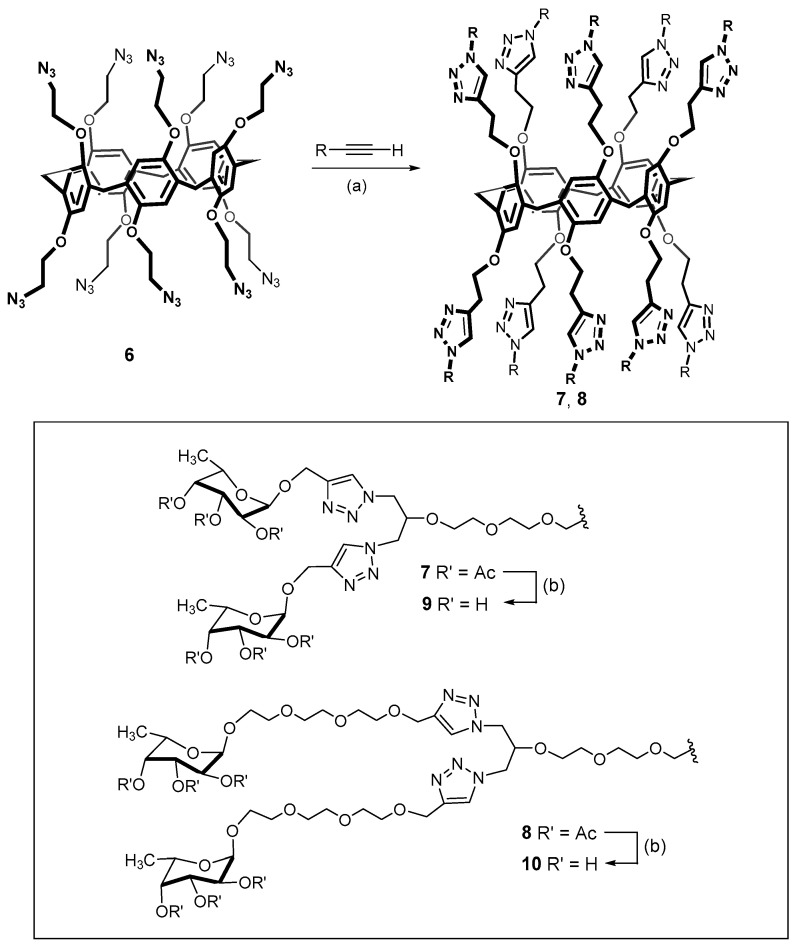
Synthesis of the first- and second-generation dendrimers with terminal sugar residues. Reagents and conditions: (a) CuI, *i*Pr_2_NEt, DMF, 110 °C, 15 min microwaves, 77% (compound **7**), 71% (compound **8**); (b) MeOH, MeONa, room temperature, 84% (compound **9**), 72% (compound **10**). Reprinted with permission from Ref. [53]. Copyright 2016 Wiley-VCH GmbH, Weinheim.

The first-generation dendrimers with peripheral sugar residues were obtained similarly [53]. However, in this case the conjugation of macrocyclic deca-azide **6** was carried out with a series of propargylated derivatives provided from the acetylated glycoclusters **7** and **8** (Scheme 2).

Initially, acetyl derivatives were obtained with yields of up to 77%. Subsequent deprotection of the acetate ester moieties led to the obtaining of water-soluble glycoclusters **9** and **10** with yields of up to 84%, which were capable of binding Pathogenic Bacterial Lectins (LecB from *Pseudomonas aeruginosa* and BambL from *Burkholderia ambifaria*). It is important to note that the obtained dendrimer derivatives go beyond the unbranched analogs with binding capacity. Increasing affinity for lectins can also be achieved by varying the length of the spacer between the carbohydrate fragments and the macrocyclic core.

**Figure 2:**
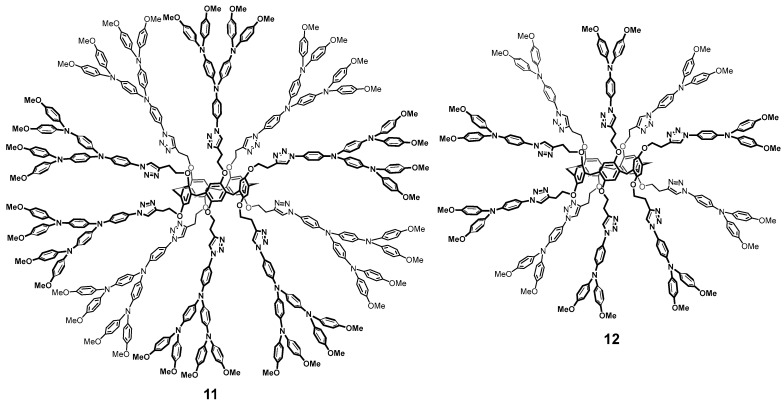
Structure of the dendrimer-like pillar[5]arene derivatives **11** and **12**.

Pillararenes are efficient electron transfer mediators [54]. Their combination with triarylamine [55] allowed the researchers to obtain first- and second-generation dendrimers **11** and **12** with satisfactory yields of 50 and 55%, respectively, (Figure 2). These multi-branched molecules were proposed as the charge transferring materials within the fields of perovskite solar cells [56]. The resulting compounds absorbed in the visible region of spectrum and the cells based on them could afford power conversion efficiency of 16.5% for the second-generation dendrimer and 10% for the first-generation ones, respectively.

### 2.2. Dendrimers Based on Resorcinarenes

**Figure 3:**
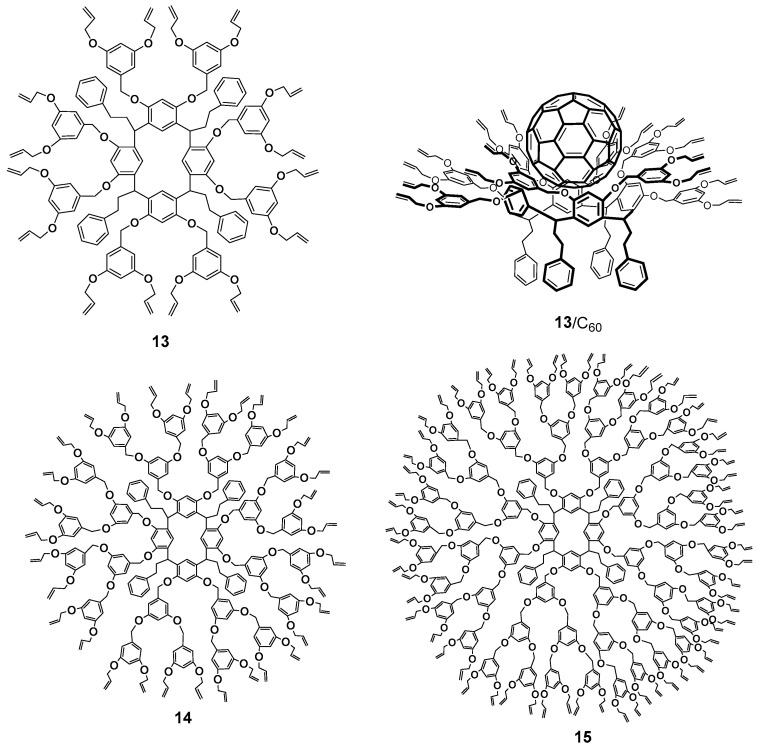
First–third generation dendrimers with terminal allyl fragments **13**–**15** and an example of the complex with fullerene **13**/C_60_.

So far, significant progress has been made in the synthesis of calixresorcinarene-based dendrimers [41]. The history of resorcinarenes comprises more than one and a half century, so all of their properties have been studied rather profoundly during this period [57,58]. The bowl-shaped form of resorcinarenes provides their rigid framework and pre-organizes the surface, giving it a concave shape. This feature became the reason for the excellent binding properties of these compounds and allowed them to imitate the molecular recognition properties of enzymes [59]. Similarly to pillararene, the macrocyclic platform of resorcinarene has chirality which appears in a number of conformers. Optical activity of the macrocycles was provided by the asymmetric bridge carbon atom [41]. Contrary to the pillararenes, the control of chiral resorcinarenes synthesis was better developed. Stepwise functionalization using chiral catalysts or solvent-controlled conformational changes is widely used for these purposes [60,61,62]. The presence of two hydroxyl groups in the resorcinol molecule allows the researchers to obtain the dendrimers with a large number of branches based on resorcinarenes, whereas the functionalization of macrocycle hydroxyl groups creates the first branching. Dendrimers of the first-third generations with terminal *n*-propyl and allyl fragments **13**–**15** synthesized on the base of resorcinarene by the convergent method were able to form complexes with fullerene C_60_ due to the implementation of strong π-π, CH-π and n-π interactions (Figure 3) [63,64].

**Scheme 3:**
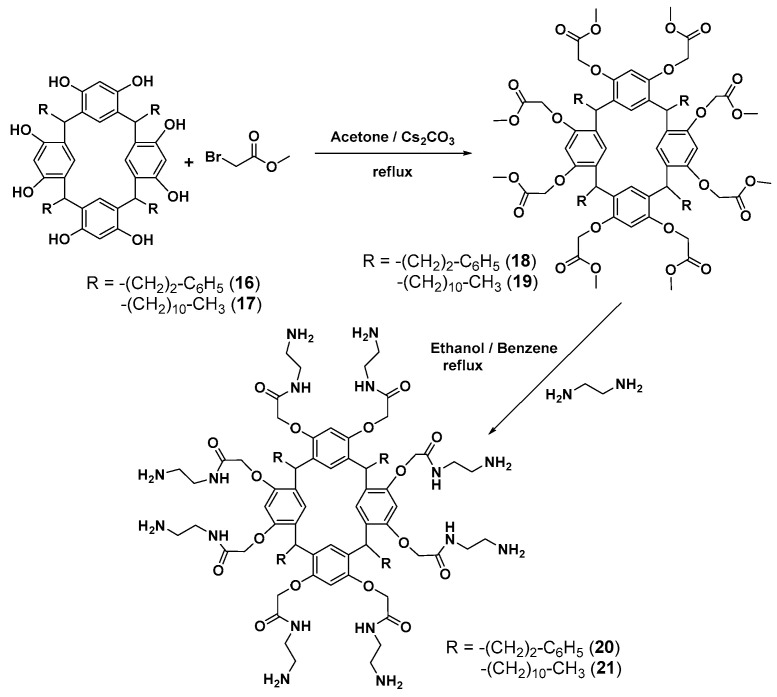
Divergent synthesis of the first-generation dendrimers based on resorcinarene.

First-generation poly(amidoamine) (PAMAM) dendrimers with a calixresorcinarene core **20** and **21** were obtained by a divergent method via the O-alkylation of resorcinarenes **16** and **17** with methyl bromoacetate followed by the treatment of the resulting products **18** and **19** with 1,2-diaminoethane (Scheme 3).

**Scheme 4:**
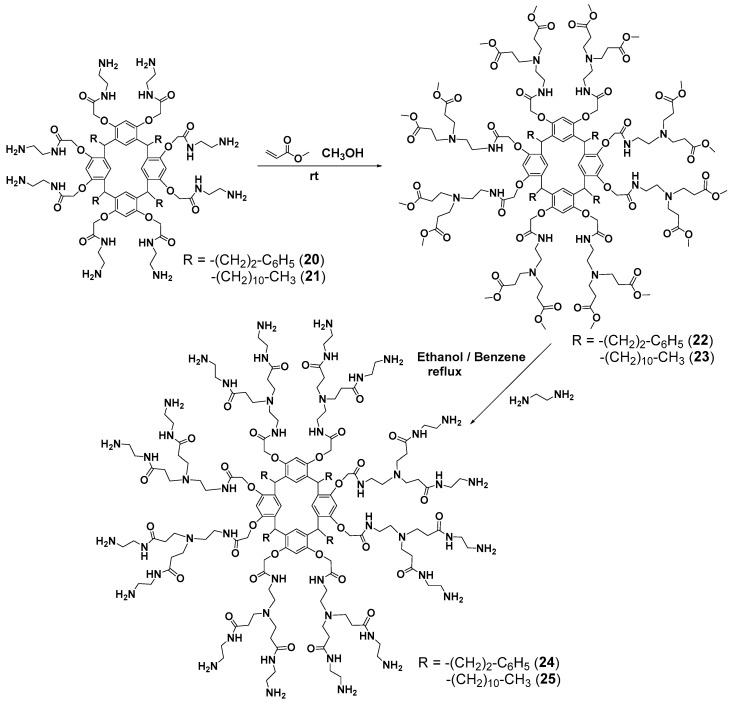
Synthesis of the second-generation dendrimers based on resorcinarene.

Branching in the second-generation dendrimers was initialized by the reaction of the amines obtained with methyl acrylate [65] (Scheme 4). The resulting PAMAM dendrimers **24** and **25** were used to impregnate a nylon membrane with a pore size of 0.45 μm which turned out to be efficient for divalent metal (Cu^2+^ and Ni^2+^) binding.

**Scheme 5:**
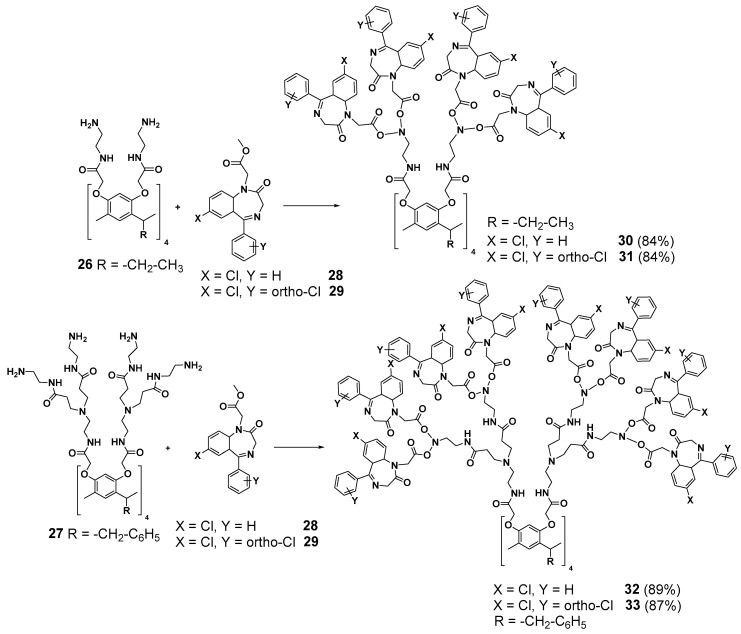
Functionalization of the resorcinarene-based dendrimers with 5-aryl-1,4-benzodiazepine moieties.

Mexican researchers have established some interesting prospects for resorcinarene core dendrimers’ application in cancer therapy. Functionalization of the terminal amino groups of the first- and second-generation PAMAM dendrimers (**26** and **27**, respectively) by 5-aryl-1,4-benzodiazepines groups (**28** and **29**) possessing anticancer activity [66] allowed to obtain **30**–**33** with good yields (84–89%). The dendrimers synthesized appeared to be efficient anticancer agents [67] (Scheme 5). Compound **31** (the first generation containing 16 diazepine fragments with a chloro-substituent in the second ring of the 5-aryl-1,4-benzodiazepine group) was found to be the most efficient agent against prostatic adenocarcinoma, colorectal adenocarcinoma and lung adenocarcinoma in humans. Reducing the chlorine atom numbers (compound **30**) led to a more than double decrease of the cytotoxicity. Interestingly, even the first- and second-generation dendrimers (**26** and **27**, respectively) without diazepine groups have demonstrated good anticancer properties.

**Figure 4:**
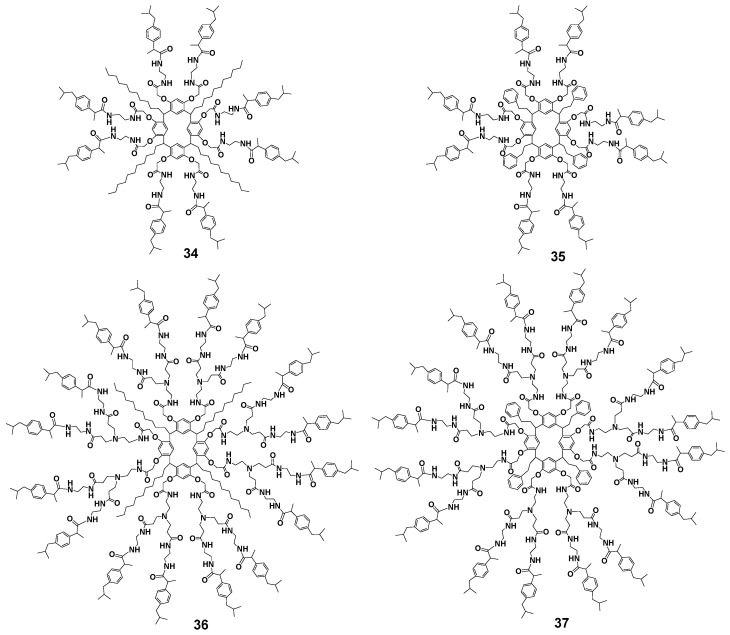
The first and second generations of dendrimers with terminal ibuprofen fragments. Reprinted with permission from Ref. [70]. Copyright 2017 MDPI.

Analogs of dendrimers **30**–**33** with ibuprofen which are other drugs with anticancer properties [68,69] were obtained by boiling ibuprofen with dendrimers containing terminal amino groups in good yields, too (70% for the second generation **36**, **37** and 90–95% for the first generation **34**, **35**, respectively, Figure 4) [70]. These compounds turned out to be almost nontoxic for healthy cells (human gingival fibroblast cell line), while being very efficient against other cancer cell lines (human glioblastoma, human prostatic adenocarcinoma, human chronic myelogenous leukemia cells, human mammary adenocarcinoma). The second-generation dendrimer with 16 ibuprofen fragments **37** bearing an aromatic substituent at the methylene carbon atom of the macrocycle had the highest selectivity towards cancer cells. Cellular internalization of the resorcinarene conjugate with ibuprofen was achieved apparently via clathrin-mediated endocytosis. The release of ibuprofen as a result of hydrolysis occurred rather slowly and strongly depended on the length of the branches in the dendrimer, reaching 80% in the case of the second generation.

**Figure 5:**
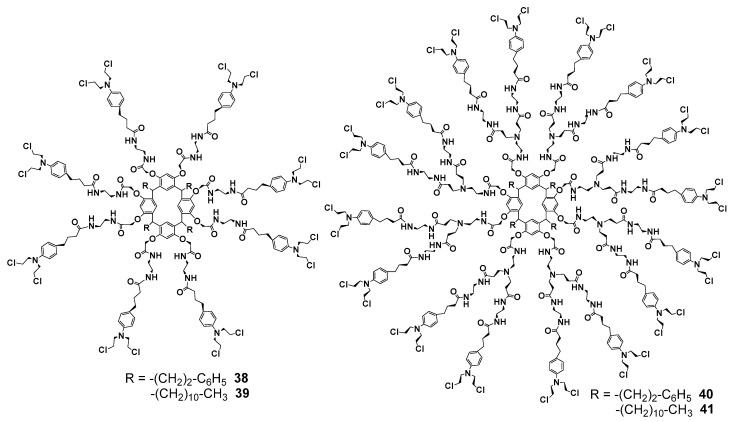
First- and second-generation dendrimers with terminal chorambucil fragments.

Chorambucil moieties were also put into resorcinarene dendrimers as terminal fragments using the methods described above (Figure 5) [71]. The first generation was obtained with good yields (71-85%), while the synthesis of the second generation was more complicated (32–52%). Such a significant difference between the yields of the first and second generations can be decreased in future, as it was obviously provided by the elimination of the protocol drawbacks. Thus, steric factors must not significantly influence the reaction process which was demonstrated in the work [67].

The resulting dendrimers **38**–**41** were efficient against human chronic myelogenous leukemia cells. Their activity was comparable to that of cisplatin, while these conjugates were less toxic towards the human gingival fibroblast cell line.

**Scheme 6:**
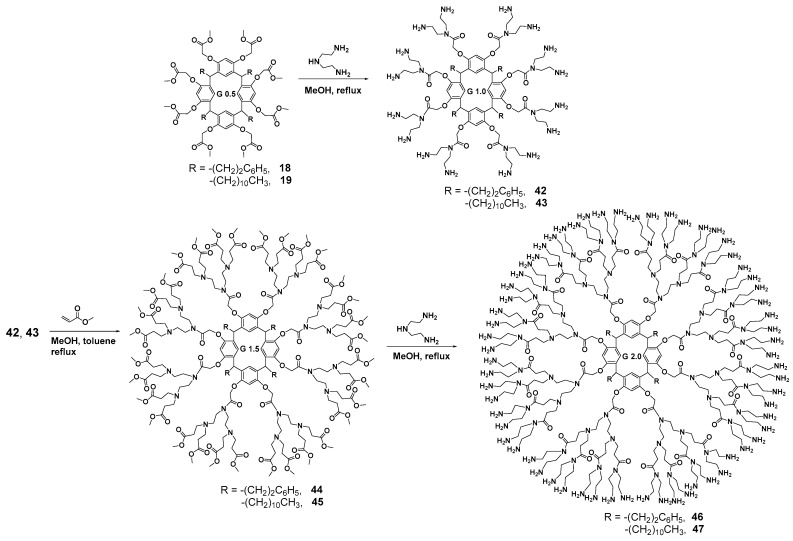
Diethylenetriamine used for resorcinarene-based dendrimer branching.

**Scheme 7:**
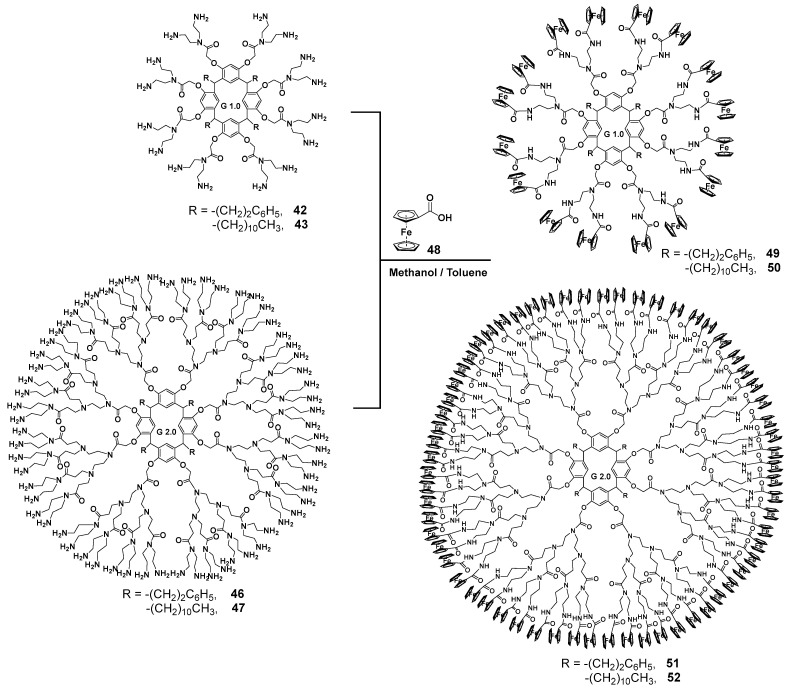
Synthesis of metallodendrimers based on resorcinarene and ferrocene carboxylic acid.

Diethylenetriamine was successfully used as an amine component [72] for creating the PAMAM dendrimer branches. This compound was used for the aminolysis of resorcinarene pre-functionalized with methyl bromoacetate at its hydroxyl groups. This method allowed the researchers to obtain first-generation dendrimers **42**, **43** with excellent yields up to 95% (Scheme 6). For second-generation synthesis, the authors used a conventional approach, namely the Michael addition of methyl acrylate in methanol, to obtain the one-and-a-half generation **44**, **45**. As a result, second-generation dendrimers **46**, **47** were also obtained by the interaction with diethylenetriamine in very good yields reaching 91%. However, the authors did not stop at these products but used them to synthesize metallodendrimers. Thus, the dendrimers obtained were involved in the reaction with ferrocene carboxylic acid **48** in methanol/benzene 1:1 at reflux (Scheme 7). The target metallodendrimers **49**–**52** were synthesized in good yields (86–91%). The authors considered the dendrimers obtained to be promising ones for optoelectronic applications, since they were capable of fluorescence in the UV and visible ranges, while their spectrum characteristics significantly depended on the number of ferrocene fragments in the molecule.

**Scheme 8:**
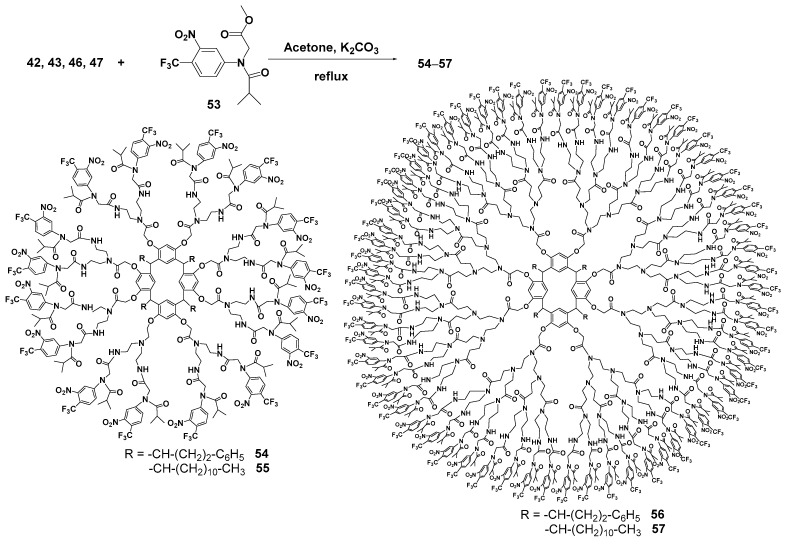
Conjugation of the resorcinarene-based dendrimers with flutamide.

Introduction of flutamide instead of ferrocene groups as terminal groups in PAMAM dendrimers with a resorcinarene core **42**, **43**, **46**, **47** [73] allowed the researchers to obtain first- and second-generation dendrimers with sufficient anticancer properties. The conjugates were obtained by introducing the flutamide ether derivative **53** to the dendrimer amino groups but compared to the ferrocene derivatives the yields of the target products **54**–**57** were lower (9–20%) (Scheme 8). The resulting conjugates (especially the second-generation dendrimer bearing 64 flutamide fragments) were efficient against three cancer cell lines, such as malignant glioblastoma, prostate adenocarcinoma and chronic myelogenous leukemia, also having low toxicity towards healthy cells. The mechanism of their action was explained by rather easy amide bond hydrolysis which provided the consequent release of the drug. It is important to note that the conjugation of flutamide with dendrimers improved its cytotoxic effect compared with unmodified flutamide, and reduced some undesirable effects.

**Scheme 9:**
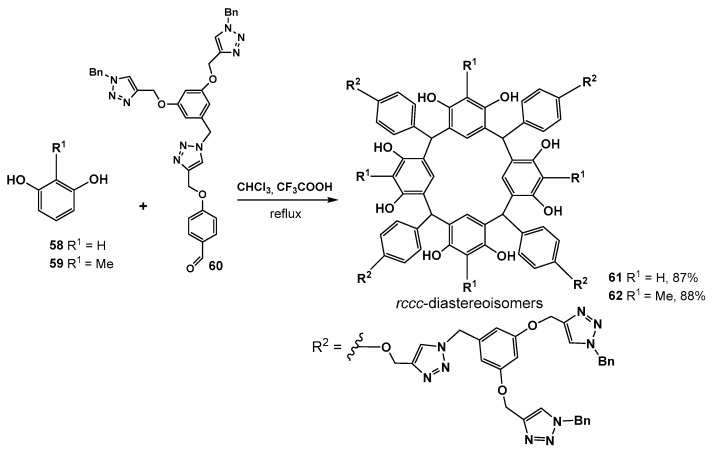
Synthesis of the dendrimers based on resorcinarenes with dendron fragments at bridging methylene groups.

Another interesting approach to the synthesis of resorcinol dendrimers was proposed by the authors of [74]. In this work, the dendron fragments were introduced to the dendrimer from the aldehyde component during the synthesis of the macrocyclic core itself. So, the resulting dendrimer had the branches linking to the core not by the oxygen atom of the resorcinol group but by a bridging methylene group (Scheme 9). The method was based on the condensation reaction of resorcinol **58** or 2-methylresorcinol **59** with the dendron **60** by boiling in chloroform in the presence of trifluoroacetic acid. Target dendrimers **61** and **62** were obtained in good yields of 87–88%.

### 2.3. Dendrimers Based on (Thia)Calixarenes

**Figure 6:**
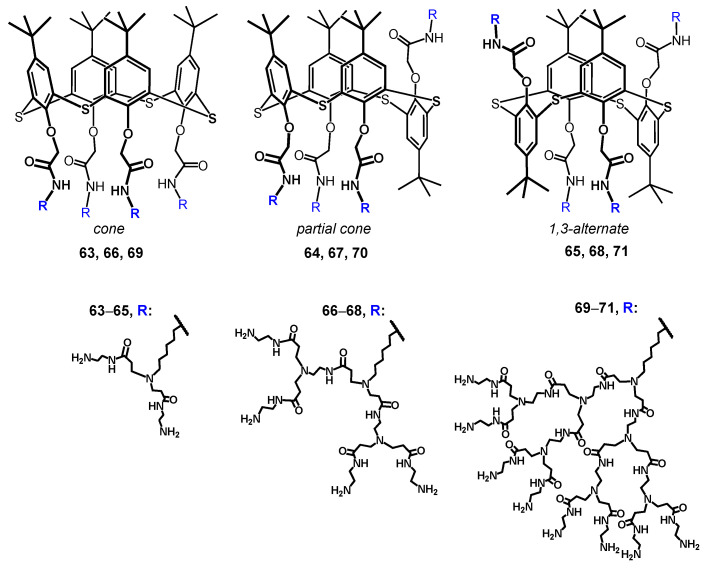
Dendrimers based on different stereoisomers of thiacalix[4]arene of first, second and third generations. Reprinted with permission from Ref. [85]. Copyright 2022 MDPI.

Today, the most thoroughly studied class of macrocyclic cyclophane dendrimers are the compounds with a (thia)calixarene core [75,76,77,78,79,80,81]. (Thia)calixarenes are the analogs of resorcinarenes with the macrocyclic platform formed with phenolic units [39,40]. They also have a bowl-shaped form and can exist in several spatial conformations, but their platform itself has no chiral properties. Nevertheless, pre-organization of the substituents around the (thia)calixarene platform and their rigid spatial localization allow them to recognize various biosystems selectively [82] including modulating enzyme activity assessment [83,84]. It is worth noting that such an effect of those (thia)calixarene derivatives without dendron fragments also remained the same in dendrimer analogs. It is interesting that the generation value does not significantly affect the binding efficiency of macrocyclic dendrimers based on thiacalix[4]arene. Thus, using the example of the hybrid PAMAM dendrimer with a thiacalixarene cycle (PAMAM-calix-dendrimers) of the first and second generations **63**–**68**, it was shown they could bind a number of catecholamines (dopamine, adrenaline and noradrenaline), while the binding efficiency was hardly dependent on generation (Figure 6). Interestingly, the monomeric analog was incapable of binding catecholamines. Thus, the template effect of the macrocycle played the key role in the formation of the complexation ability of PAMAM-dendrimer hybrids with a thiacalixarene macrocyclic fragments, which allows the binding groups re-localization to implement the multivalent effect [85]. The most significant contribution to the complexation process was made by the H-bonds formation, as well as van der Waals forces. In addition, the binding was affected by the size of internal cavities in the dendron fragments. Presumably, the complexation was predominantly carried out by the amino groups located in the internal cavities of the dendrimers, rather than the terminal ones. It was evidenced by both the data of two-dimensional ^1^H-^1^H NOESY NMR spectroscopy and the absence of noticeable affects of dendrimer generation on the binding efficiency. Previously, the macrocyclic platform of thiacalix[4]arene was shown to contribute to hormone-binding significantly [86].

**Figure 7:**
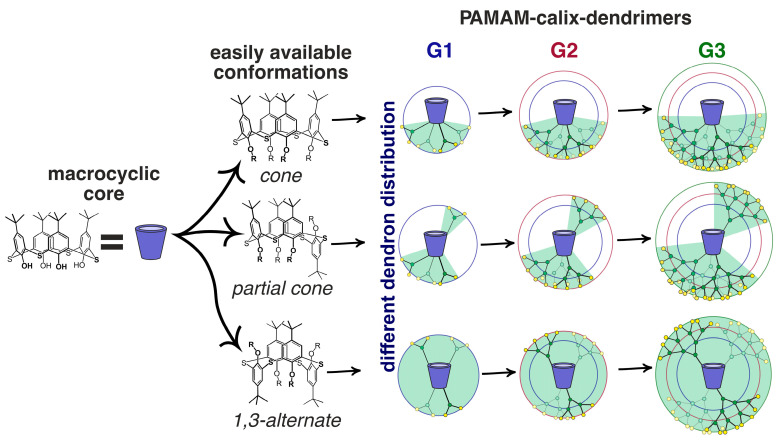
Charge distribution in PAMAM-calix-dendrimers relative to the macrocyclic platform. Reprinted with permission from Ref. [87]. Copyright 2024 MDPI.

The (thia)calixarene platform advantages for the design of dendrimers appeared as unexpected decreasing of the hemolytic activity of dendrimers with the growth of generation [85,87], which fundamentally distinguishes them from the conventional dendrimers [88,89]. In case of the thiacalixarene core, the hemolytic activity strongly depends on the macrocycle conformation having maximum value for the *cone* stereoisomer (**63**, Figure 6). This can be explained by the higher density of the cationic charge, which is distributed unevenly throughout the nanoparticle and accumulated on one side of the macrocyclic platform. Consequently, the enhancement of negative effects when interacting with red blood cells took place. *1,3-alternate* Stereoisomer **71** (*1,3-alternate)* has the least hemolytic activity, as the charge density is the most uniformly distributed in its associates (Figure 7). The work [87] described the inability of third generation dendronized thiacalix[4]arenes **69**–**71** to induce platelet aggregation: apar from having no interaction with platelets, they also did not hinder the contribution of all coagulation factors.

**Figure 8:**
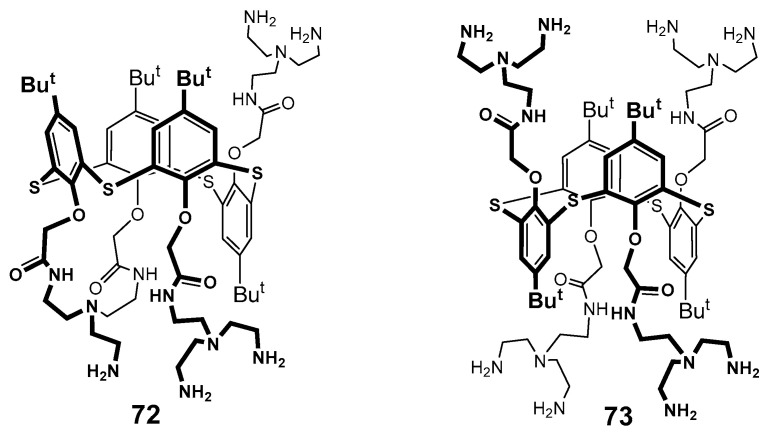
Dendronized derivatives of *p-tert*-butylthiacalix[4]arene **72** and **73**.

It is well known that dendrimers are capable of binding biopolymers, in particular DNA. As a rule, only conventional high generation dendrimers which are usually cytotoxic can bind DNA. The choice of thiacalixarene core as the platform has allowed researchers to bind DNA from salmon sperm and calf thymus with the first generation already (**63**–**65**, **72**, **73**, Figure 6 and Figure 8) [90,91,92].

**Figure 9:**
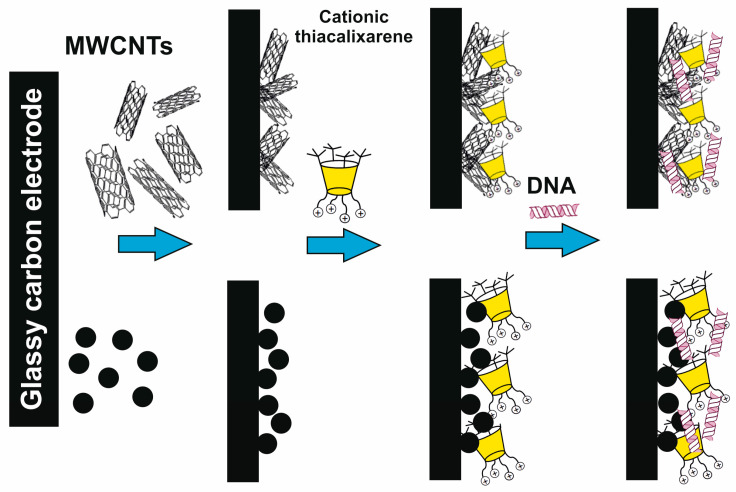
Schematic representation of the operating principle of electrochemical DNA sensors based on dendronized thiacalixarenes for doxorubicin determination. Reprinted with permission from Ref. [96]. Copyright 2023 MDPI.

**Figure 10:**
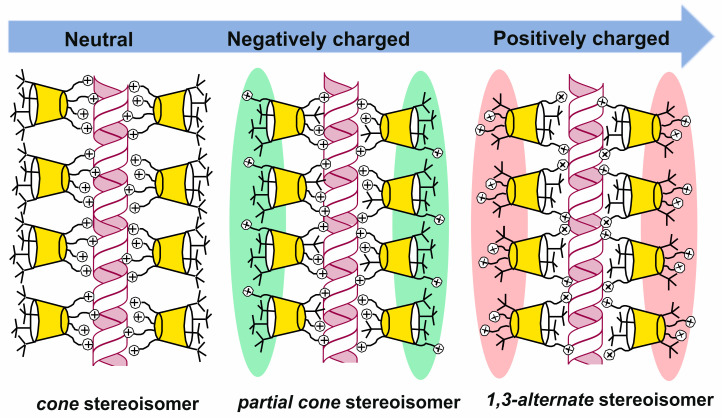
Schematic representation of different charge formations at the surface of electrochemical DNA sensors based on dendronized thiacalixarenes. Reprinted with permission from Ref. [96]. Copyright 2023 MDPI.

It was interesting that first-generation dendrimer **63** with a core of *cone* conformation, as a result of binding the biopolymer, has exerted the effect of stabilization preventing the biomolecule degradation in buffer solution. Such a core conformation effect was suggested to occur due to the isolation of the lipophilic (hydrophobic) part of the biopolymer amphiphilic compound from the aqueous solution. There was no such feature observed for the other conformations (*partial cone* and *1,3-alternate*, **64** and **65**) [90]. Interestingly, the complexation of calix-dendrimers **65**, **68** and **71** based on the macrocyclic core in *1,3-alternate* conformation with small interfering RNAs (siRNAs) protected siRNAs from degradation in the presence of RNases and serum proteins [93]. The resulting positively charged complexes were often used to deliver genetic material into cancer cells, increasing the cellular take-up of siRNAs significantly [94]. This ability of calix-dendrimers may find application in new anticancer drug designs. With generation growth, the binding abilities towards the biopolymers (namely DNA) certainly increased [87]. In this case, the macrocyclic platform conformation influencing the binding process has almost disappeared. The secondary structure of DNA remained unchanged during the interaction with all conformational isomers of PAMAM-calix-dendrimers. The interaction of calix-dendrimers with DNA did not limit the access of small molecules to the biopolymer. Thereby the process of their binding was used as a platform for the electrochemical DNA sensor which allowed the determination of the intercalator drug doxorubicin with a limit of detection equal to 10 pM [95] (Figure 9). The widening of the PAMAM-calix-dendrimer range was investigated and the analysis conditions optimization allowed researchers to improve the detection limit up to 1 pM [96]. These sensors contained the assembly of thiacalix[4]arene-based dendrimers in *cone*, *partial cone*, or *1,3-alternate* conformation on the glassy carbon electrode surface coated with carbon black or multiwalled carbon nanotubes, where the DNA layer was additionally applied via electrostatic interactions (Figure 10). Choosing the conformation of the macrocyclic core has allowed researchers to form differently charged complexes with DNA (positive, negative and neutral), which can be used to determine various types of low-molecular substrates.

**Figure 11:**
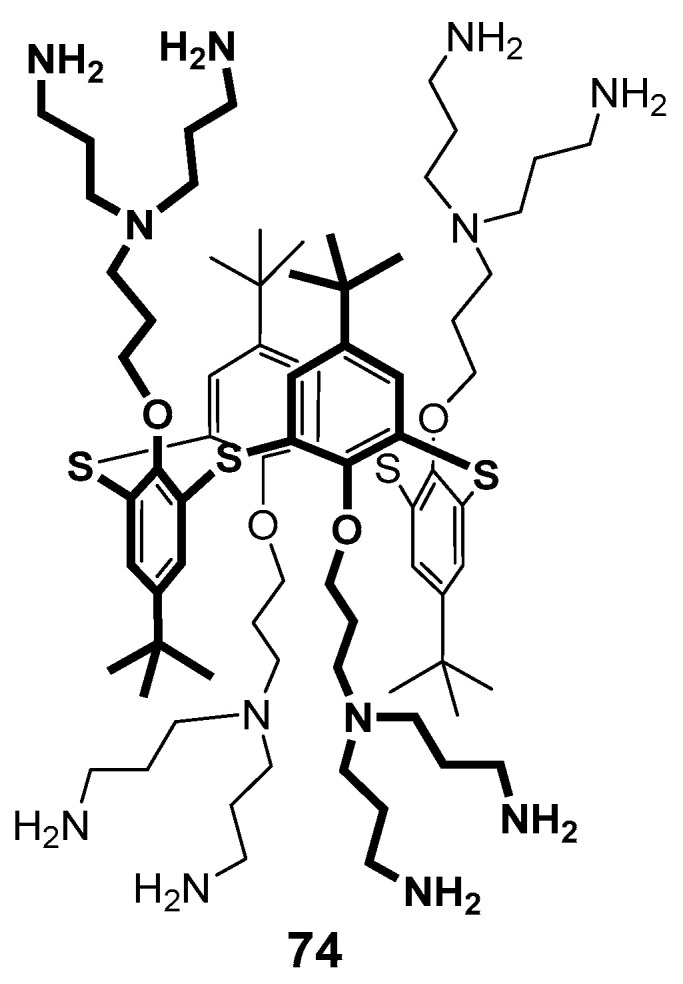
Dendronized thiacalixarene **74**.

The structure of the stable complex of thiacalixarene **74** containing terminal ammonium groups with double stranded palindromic DNA decamer d(GCGTTAACGC)2 was determined using molecular docking and NMR spectroscopy methods (Figure 11) [91]. This decamer was chosen as a model compound to simplify the spectral pattern. It turned out that thiacalixarene predominantly interacted with the central fragment of the decamer, mainly to the thymine fragment, and the *tert*-butyl protons of the dendrimer were brought close to the methyl group of thymine. Two variants of the complexes were considered using the molecular docking method, such as binding via the major and minor grooves of the oligonucleotide. Despite the values being close to each other for both the complexes, the authors preferred the interaction via the major groove, since this type of complex was in better agreement with the NOESY NMR spectroscopy data.

**Figure 12:**
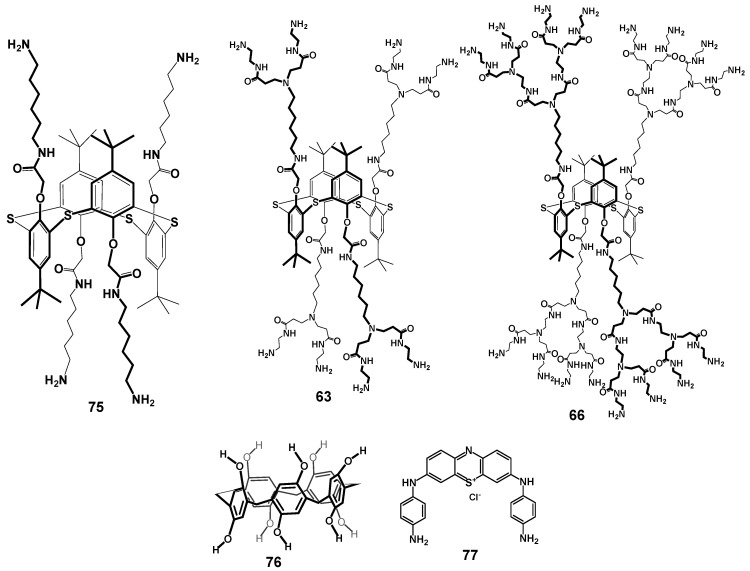
Components of the electrochemical biosensor for the determination of uric acid. Reprinted with permission from Ref. [54]. Copyright 2024 MDPI.

The combination of zero-second-generation PAMAM-calix-dendrimers **75**, **63**, **66** with pillararene **76** (efficient electron transfer mediator) and phenothiazine derivative **77** was used as the platform for electrochemical biosensor development (Figure 12) [54]. The role of calix-dendrimers in the sensor assembly was to increase the efficiency of phenothiazine derivative electropolymerization, as well as to prevent the inactivation of pillararene due to its oxidation product accumulation on carbon black surface. This biosensor allowed researchers to determine uric acid in the concentration range of 10 nM–20 μM with a limit of detection of 4 nM. It was shown that the determination was not interfered with by such components as dopamine, glucose, and ascorbic acid. The authors found that using higher (second) generation dendrimers made it possible to increase the currents recorded, thus enhancing the sensitivity of the sensor.

**Figure 13:**
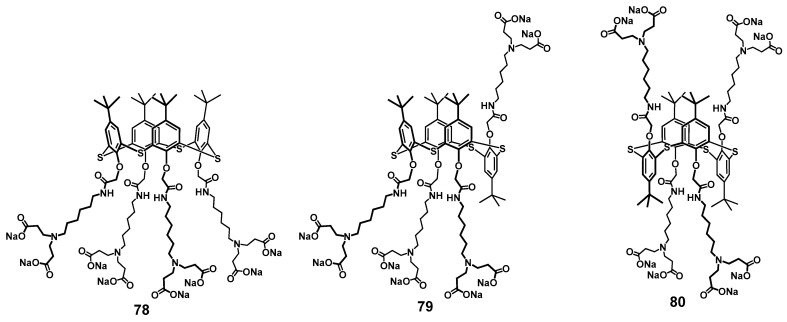
Calix-dendrimers with terminal carboxylate fragments capable of inhibiting lysozyme fibrillation. Reprinted with permission from Ref. [100]. Copyright 2024 MDPI.

In addition to DNA, PAMAM calix-dendrimers can bind the proteins effectively. Thus, binding of dendrimers **63**–**65** of the first generation with chicken egg lysozyme led to the formation of supramolecular complexes which enhanced the antibacterial action of the enzyme [97]. Interestingly, those classical PAMAM dendrimers with linear ethylenediamine core were capable of significant binding to lysozyme only for the third generation. It is worth noting here that using a conventional fifth-generation PAMAM dendrimer (with a 1,4-diaminobutane core, carbomethoxypyrrolidone-terminated) also provided the increasing of the antibacterial effect of histidine kinase inhibitor peptide [98] against a methicillin-resistant strain of *Staphylococcus aureus* [99]. When the ethylenediamine terminal fragments of the first-generation calix-dendrimers were replaced by anionic carboxylate fragments (compounds **78**–**80**), the ability to bind to lysozyme remained the same, and the macrocycle could stabilize the protein structure and inhibit its fibrillation [100] (Figure 13). The resulting associates had a nano- or submicron size, the minimal size was for the *partial cone* core conformation (84 nm) and the maximal for the *1,3-alternate* (682 nm), respectively. The positive charge of the particles formed allowed the researchers to suggest the protein was located at their surface. Electron microscopy methods demonstrated the difference in the morphology of the associates definitely. In case of *partial cone*, there were small spherical particles at the images, while for *1,3-alternate,* extended formations were observed representing the conglomerates of smaller particles.

**Figure 14:**
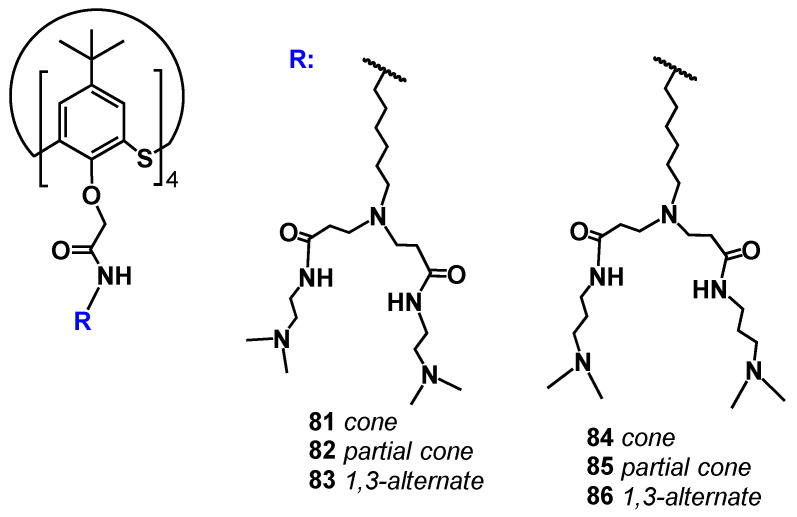
PAMAM-calix-dendrimers capable of inhibiting cholinesterase.

Due to the functionalization of the thiacalixarene backbone with fragments structurally similar to choline, the authors [83] achieved PAMAM-calix-dendrimers binding to cholinesterase (butyryl- or acetyl-) which led to the inhibition of the enzyme (Figure 14).

**Figure 15:**
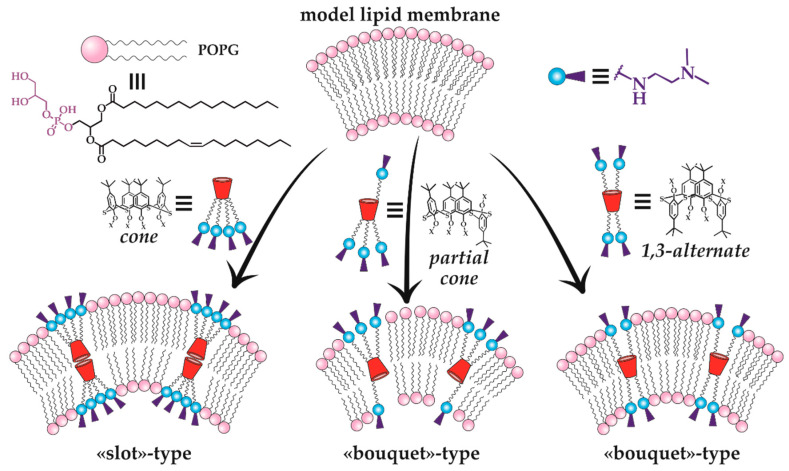
Illustration of the mechanism of calix-dendrimer different conformations’ effect on a model lipid membrane. Reprinted with permission from Ref. [104]. Copyright 2023 MDPI.

Thus, the therapeutic effect of the new macrocyclic dendrimers **63**–**65**, **81**–**86** can be expected towards Alzheimer’s disease and other neurodegenerative disorders [101,102]. Moreover, the therapeutic potential of calix-dendrimers is not limited by enzyme inhibition. Metacyclophanes are known as antibacterial agents [103] due to which their dendrimer derivatives also exhibited bactericidal properties [97,104]. It should be noted here that the bactericidal activity of PAMAM-calix-dendrimers was significantly higher than that of their traditional analogs with a linear core, as well as compared to monomeric analogs without the macrocyclic platform. If the dependence of antibacterial activity on the macrocyclic core conformation is taken into consideration, a definite pattern can be observed: the most active conformations were the *cone* and *partial cone*, in which the charge density on the particle surface was higher than the one of the *1,3-alternate*. Thus, these conformations had the most effect on the bacterial cell membrane which led to its destruction. Additional experiments with model membranes confirmed the proposed mechanism. There was the greatest change in the model vesicles’ size for the *partial cone* observed, indicating their loosening in the presence of calix dendrimer (Figure 15). The greatest activity was specific for calix dendrimers bearing terminal tertiary amino groups [104].

A distinctive feature of calix-dendrimers is the first generation exhibiting the properties of standard thiacalixarenes, such as increasing the amphiphilicity of their structure in the range 1*,3-alternate* < *partial cone* < *cone*. Due to this property, the water solubility is usually maximum for the stereoisomer with *cone* conformation of the core [97]. For higher generations, the difference between various conformations was erased due to the shielding of the macrocyclic core by the branches. In general, the analysis of all currently available data on PAMAM-calix-dendrimers allowed the researchers to conclude that it was the dendrimer with the core in the *cone* conformation that formed self-associates with minimal (nanometer) size [85,105]. The other conformations (especially *1,3-alternate*) form extended vesicles in solutions due to the branches placed at both sides of the macrocyclic core [106]. However, binding to various molecules, including biopolymers, usually led to the decreasing of the associates’ size compared to individual compounds [87,90,97]. This feature of calix-dendrimers makes them excellent candidates for drug delivery, since the small size of the complex is critically important for these purposes [107,108]. Small particles (30–200 nm) are not attacked by the body’s immune system, while they are large enough to be not excreted from the body by the kidneys. The sizes of calix-dendrimers, even in lower generations due to the presence of a macrocyclic platform, are comparable to the sizes of conventional high-generation dendrimers. Thus, the first generation of PAMAM-calix-dendrimers size corresponds to the fourth generation of PAMAM dendrimers with an ammonium core one [109,110]. The successful drug releasing from the complexes has been demonstrated using catecholamines as the example [85], which also confirmed the prospects of PAMAM-calix-dendrimers for drug delivery.

**Figure 16:**
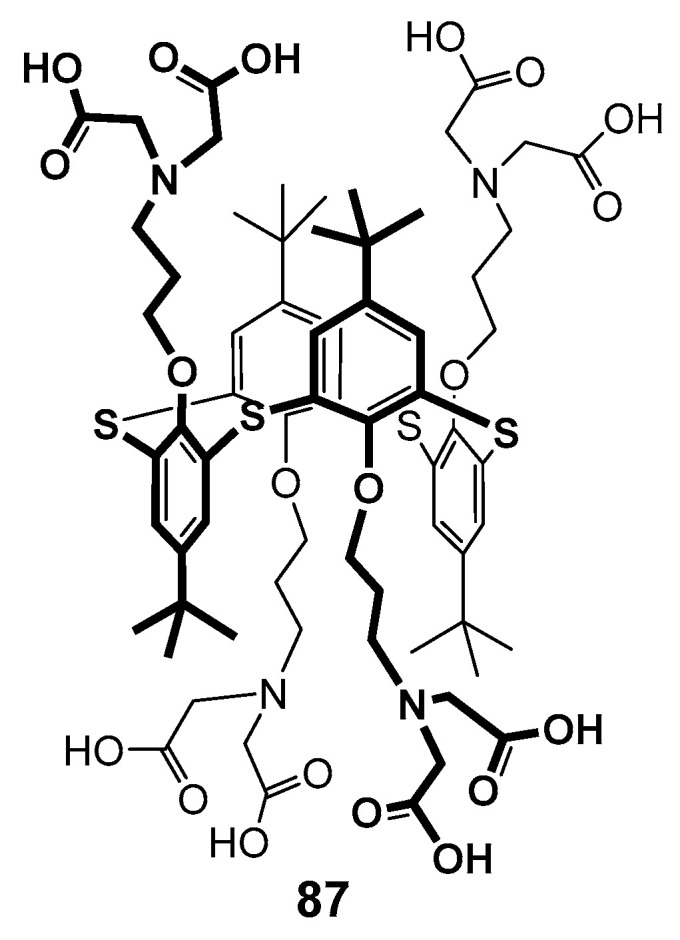
First-generation calix-dendrimer with terminal amino diacetate fragments.

In 2009, the first-generation polyamine dendrimer derivative **74** based on *p-tert*-butylthiacalix[4]arene was presented, in which the branches were formed from *N,N*-dipropyliden-amine [110,111]. Its analog **87** with amino diacetate fragments outside was also obtained (Figure 16). The effect of both compounds on the dynamic structure of liposomal membranes made of dipalmitoyl phosphatidylcholine was studied. The authors have found the binding of calixarenes occurred predominantly in the polar region of the bilayer. The hydrophilic **74** interacted with phosphate groups. It was located close to the bilayer surface and had no visible effect on the transition temperature. At the same time, dendrimer **87** with terminal amino diacetate fragments formed the bridges between positively charged choline groups of neighboring lipid molecules at the bilayer surface, thereby promoting their denser packing and increasing the phase transition temperature.

**Scheme 10:**
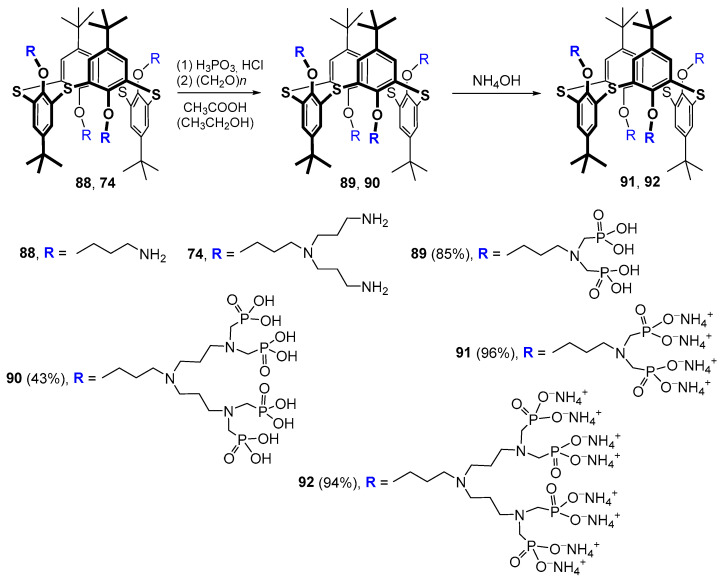
Synthesis of first- and second-generation phosphorylated calix-dendrimers by the Moedritzer–Irani method.

The phosphorylation of terminal amino groups **74** and **88** by the Moedritzer–Irani method allowed the researchers to obtain first- and second-generation dendrimers based on thiacalixarene in the *1,3-alternate* conformation with phosphonic acid fragments at the rim [112]. The following treatment of these products with aqueous ammonia led to the appropriate ammonium salts in excellent yields (94–96%) (Scheme 10). The phosphorylation itself proceeded in a good yield of 85% in the case of the first generation (compound **89**); however, for the second generation, the results were significantly worse (43%), which was apparently because of a large loss of the target product **90** due to its high solubility in water.

**Figure 17:**
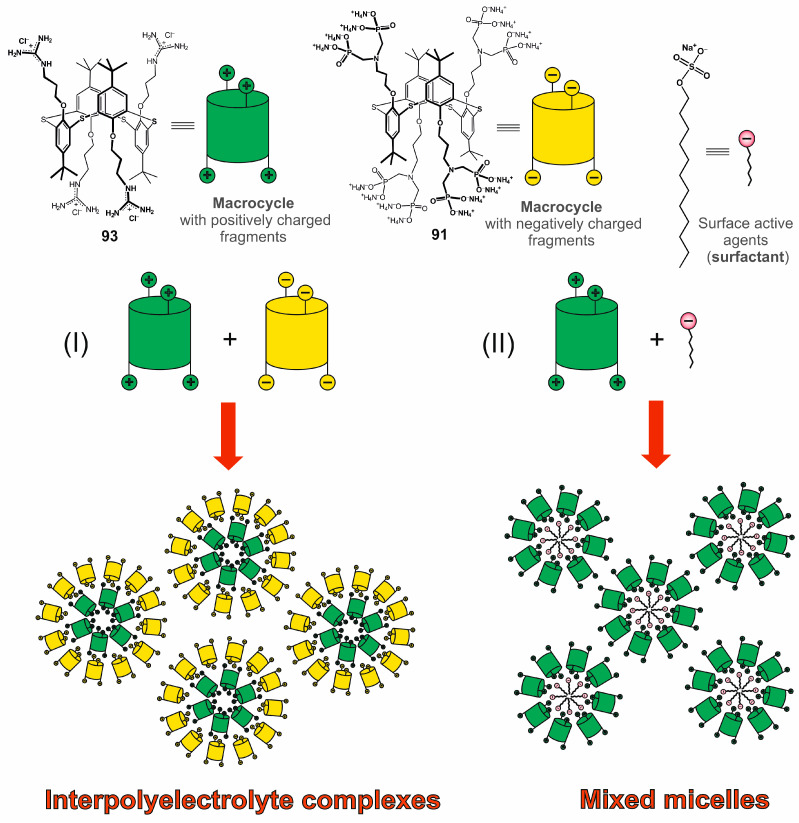
Creation of intermolecular associates based on macrocyclic compounds.

Negatively charged first-generation macrocyclic **89** and **91** formed polydisperse associates in aqueous solution [113], which were found to bind dodecyl trimethyl ammonium chloride. In this case, polydisperse systems consisting of predominantly submicron-sized particles were also formed [114]. The guanidine derivative of thiacalix[4]arene **93** was chosen as the positively charged component in inter-polyelectrolyte complexes based on **89** and **91** (Figure 17). These complexes had lower polydispersity in solution, as well as nanometer sizes of the formed particles [113]. Also, the systems formed by **89** and **91** were stabilized in the presence of silver ions, which induced reversible association/dissociation of supramolecular aggregates [115]. The described inter-polyelectrolyte particles could bind lysozyme without changing its conformation.

**Scheme 11:**
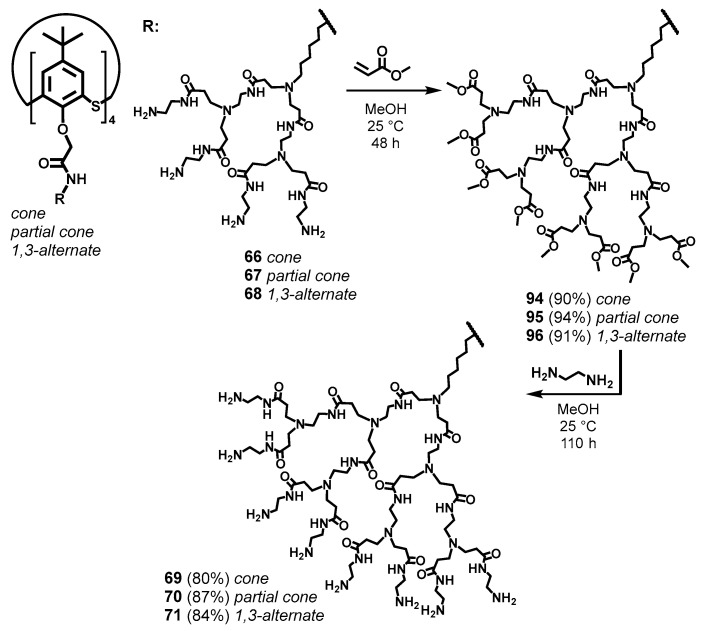
Divergent synthesis of third-generation PAMAM-calix-dendrimers. Reprinted with permission from Ref. [87]. Copyright 2024 MDPI.

**Scheme 12:**
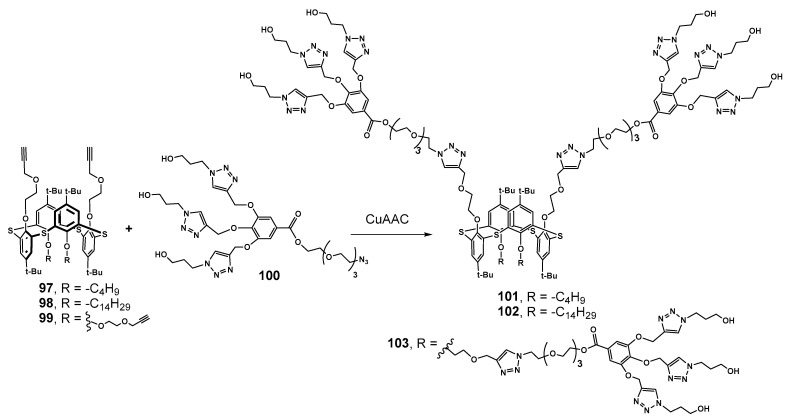
Convergent synthesis of first-generation calix-dendrimers via Cu-catalyzed alkyne-azide cycloaddition reaction.

For the thiacalix[4]arene derivatives, a divergent approach to synthesis with sequential increasing of the dendron length has been demonstrated to be excellent and allowed the researchers to obtain the target compounds in high yields [87] (Scheme 11). Amine derivatives of thiacalix[4]arene in various conformations were chosen as the cores. Methyl acrylate was used to initiate branching. It is important to note here that the key condition for the successful synthesis of target PAMAM-calix-dendrimers was the length of the aminoalkyl substituent of the core. Thus, with the length increasing, steric limitations were reduced, the probability of intra- and intermolecular cross-linking decreased, and the yields of target compounds increased significantly [116]. In the authors’ opinion, the optimal substituent in this case was hexamethylene spacer [86]. So-called half-generations **94**–**96** obtained were converted into target compounds by aminolysis reaction. The authors of [117] had limited their investigation by the first-generation dendrimers with a single conformation of the thiacalixarene core (*1,3-alternate*). A completely different approach was used for their synthesis, such as the azide–alkyne click reaction (Scheme 12).

Di- and tetrapropargyl derivatives of **97**–**99** were functionalized with previously formed gallic acid-based dendrons **100** by means of Cu-catalyzed azide–alkyne cycloaddition reaction (Scheme 12). As a result, symmetrical **103** and two amphiphilic dendrimers **101** and **102** were obtained with different lengths of the alkyl chains. Using of the convergent approach turned out to be very efficient, as the yields reached 65–89%, apparently due to the fairly free arrangement of the propargyl substituents in the *1,3-alternate* conformation. All the dendrimers obtained were capable of submicron associates’ formation in water, and the most compact particles were formed by more lipophilic compounds due to the efficient hydrophobic interactions’ contribution. Dendrimer **103** with four dendrons was the largest one and formed the extended structures, which was in good agreement with the behavior of PAMAM-calix-dendrimers of *1,3-alternate* conformation. All the dendrimers were able to bind the organic hydrophobic dye Orange OT. The highest solubilizing capacity was shown for amphiphilic **101** with butyl substituents, which provided sufficient loosening of associates. As a result, it allowed the dye to be incorporated into the hydrophobic layer of the aggregates. The ability to form nanoassociates was used to stabilize Pd nanoparticles in the Suzuki model cross-coupling reaction and to reduce *p*-nitrophenol. All dendrimer-stabilized Pd nanoparticles showed high catalytic activity. The stabilization occurred, apparently, due to the nanoparticles located in the dendrimer cavities, and their aggregation was prevented [8].

**Scheme 13:**
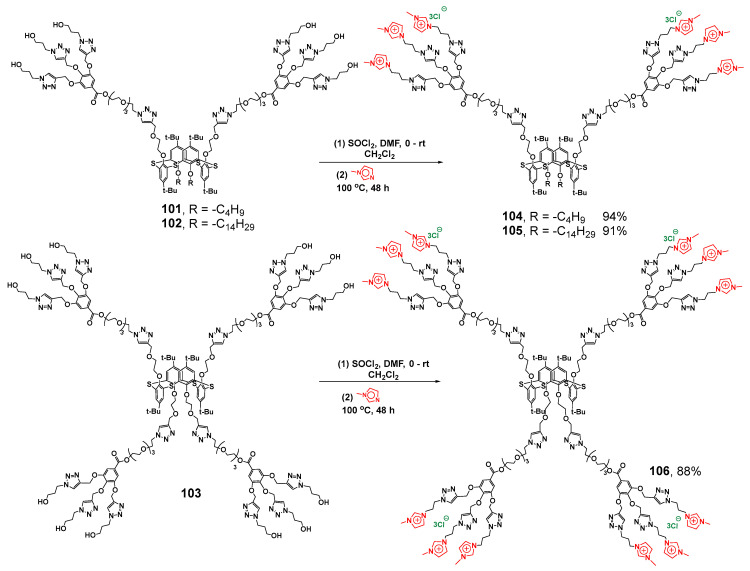
Synthesis of first-generation calix-dendrimers with terminal imidazolium groups.

The introduction of imidazolium groups as terminal ones (Scheme 13) did not cancel the ability to bind the Orange OT dye, and also unchanged the catalytic activity of Pd nanoparticles for the reduction in p-nitroaromatic compounds [118]. Positively charged imidazolium groups provided new dendrimers **104**–**106** with the ability to bind calf thymus DNA, which resulted in DNA compaction (with the maximum for symmetrical dendrimer **106** with four dendrons up to 100 nm). Binding occurred inside the aggregates via an electrostatic mechanism proved by recharging negative ctDNA to a positively charged one.

**Scheme 14:**
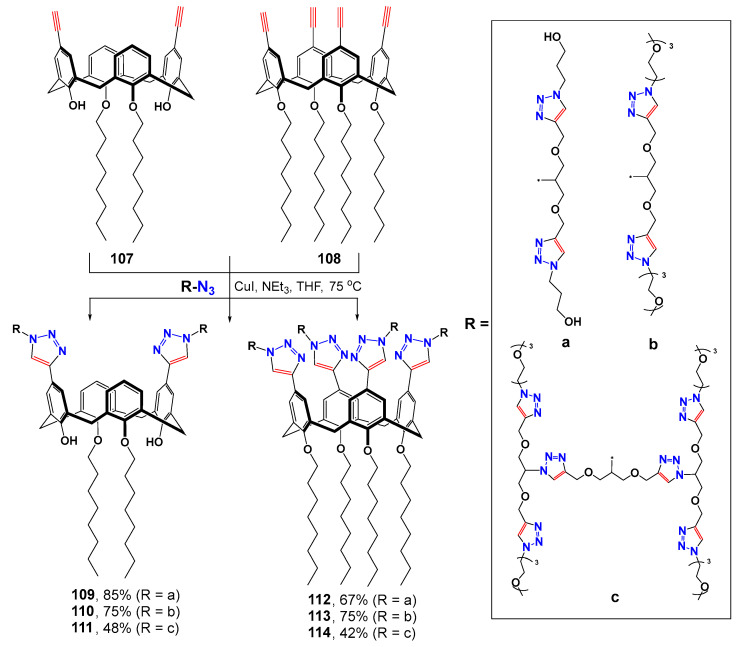
Synthesis of first-generation calix-dendrimers with terminal imidazolium groups.

The convergent synthesis method was successfully extended to the classical analog of thiacalix[4]arene—calix[4]arene (Scheme 14) [119]. The CuAAC reaction method was used to obtain dendrimers of the first (**109**, **110**, **112** and **113**) and second (**111** and **114**) generations in satisfactory yields reaching 85% for the first and 48% for the second generations, respectively. All the dendrimers obtained formed nanometer associates in water with the constituent particles of 80–130 nm. The greater the number of hydrophilic substituents (dendrimers with four branches), the larger the size of the particles formed. The dendrimers obtained could also stabilize palladium nanoparticles which were subsequently used as the catalysts in the cross-coupling reaction between *p*-bromoanisole and phenylboronic acid in water. The stabilization of metal nanoparticles occurred due to their agglomeration hindrance when they were bound to dendrimers. Dendrimer-stabilized nanoparticles had lower catalytic activity compared to free nanoparticles and to stabilized PEH-16 polyol. The best conversion was observed in the case of more branched tetra-alkyl substituted calixarenes and at the concentrations above the critical micelle concentration (CMC) value. Dendrimers with oxyethyl substituents exhibited the highest selectivity, while hydroxydendrimers were significantly way below them in this regard. Side biphenyl was formed in their presence. Dendrimers with concentrations above their CMC also turned out to be efficient in the reaction of *p*-nitrophenol reduction. Stabilization of palladium nanoparticles with dendrimers was also used for the reduction in hydrophobic *p*-ethylnitrobenzene which, unlike *p*-nitrophenol, was practically insoluble in water. In this case, the second generation branched dendrimers showed worse results compared to the first-generation analogs due to the bulk hydrophilic branches preventing the metal nanoparticles from their approach close to the solubilized hydrophobic substrates.

**Scheme 15:**
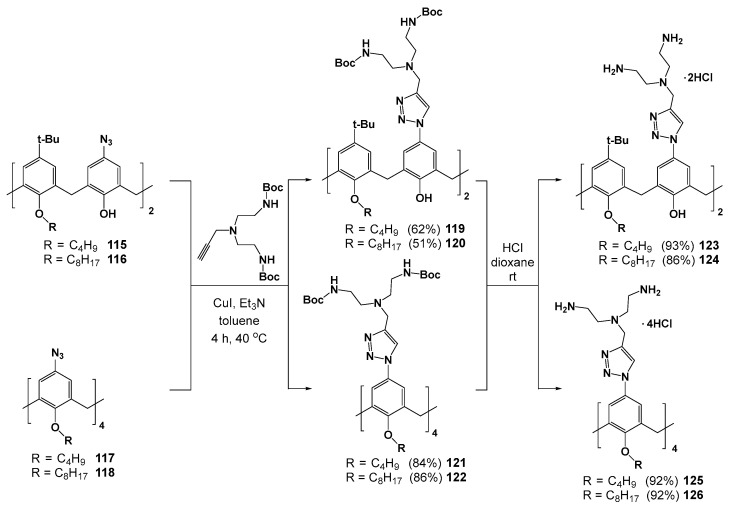
Dendronization of the upper rim of thiacalixarenes.

Dendrimers **123**–**126** with terminal ammonium groups at the upper rim were obtained by similar click reaction method using azide derivatives of calix[4]arene **115**–**118** and propargylamines (Scheme 15) [120,121]. The resulting compounds have formed positively charged submicron associates in water (electrokinetic potentials of +40 ÷ +60 mV) with CMC significantly depending on the compound hydrophobicity. The replacement of two polar ammonium groups with *tert*-butyl groups (compounds **123** and **124**) at the upper rim led to the decreasing of CMC from 790 to 5 μM [121]. The aggregates of **123**, **124** and **125**, **126** were able to bind negatively charged eosin Y dye, as they maintained a positive charge. The resulting complexes were used as the nucleotide probes whose action was based on competitive substitution of the dye for nucleotide. Interestingly, the most efficient binding was observed for disubstituted **123**, **124** with the less charged adenosine-5’-diphosphate (ADP), rather than with adenosine-5’-triphosphate (ATP). The authors explained this behavior with a quantum chemical approach. The significantly larger size of ATP compared to ADP did not allow the former to achieve good geometrical coincidence with two ammonium fragments of the receptor. The method developed has provided the visual detection of ADP with a limit of detection equal to 0.5 mM.

**Figure 18:**
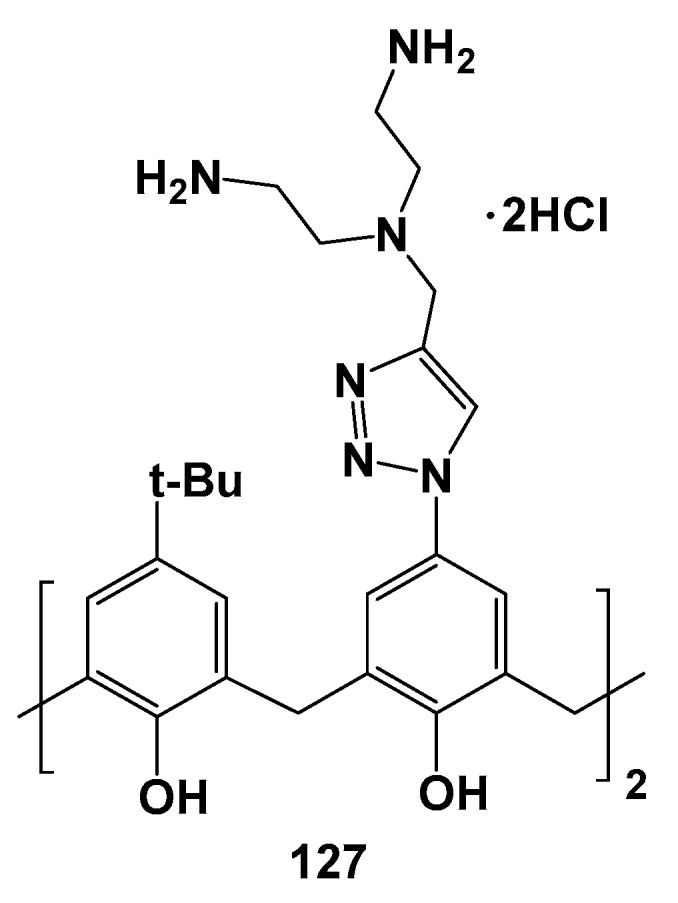
Polyammonium compound **127**.

The trend of the CMC decrease with growing hydrophobicity of the compounds also remained in the series of **125**–**127** substituted with ammonium fragments at the upper rim (Scheme 15, Figure 18) [120]. For the most hydrophilic **127** with phenolic groups at the lower rim, the CMC was not determined. When phenolic groups were replaced by ethyl and tetradecyl fragments, the CMC reached 5.0 and 3.4 μM, respectively. In this case, the associates of the minimum size were formed for the most hydrophobic **126** (19 nm), while for **125** they had already become significantly larger (145 nm). All the dendrimers studied could bind calf thymus DNA by the intercalation mechanism, which was proved by the significant increase in DNA melting temperatures in their presence. The greatest increase in temperature was achieved for **126** (from 72.5 to 84.2 °C). Dendrimers binding with DNA led to its compaction which also turned out to be maximum in the case of **126** (22 nm).

**Scheme 16:**
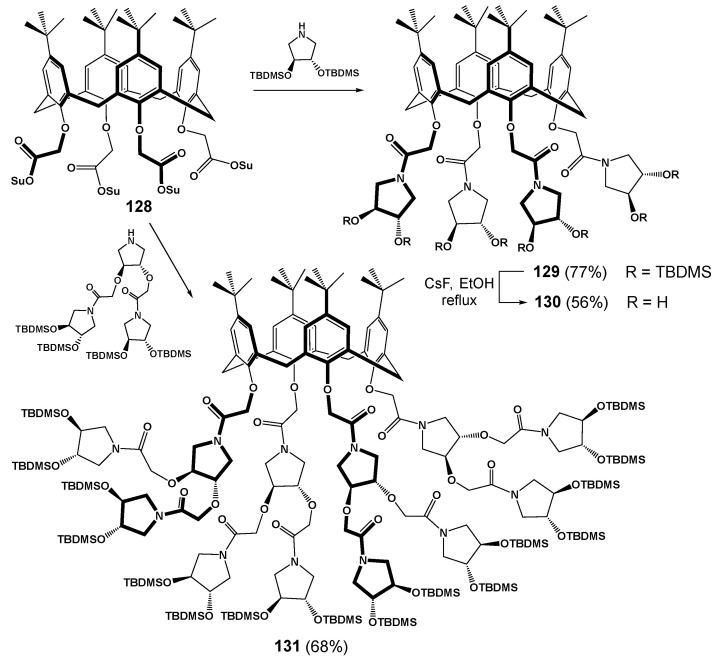
Synthesis of calixarene-based dendrimers **129** and **131**. Reagents and conditions: DIPEA, CH_2_Cl_2_, 30 °C, 5 d, (Su: succinimidyl; TBDMS: *tert*-butyldimethylsilyl).

The convergent synthesis method was also used to obtain some derivatives of classical calix[4]arene of *cone* conformation but there was no possibility to obtain any generations higher than the second one (Scheme 16) [122]. It should be noted that the yields of the target products were significantly lower than in the case of thiacalixarene (77% for the first generation (**129**), 68% for the second generation (**131**), respectively). Obviously, this was due to the smaller size of the classical platform and steric limitations of its functionalization. The fact that the authors chose the convergent route of functionalizing of rather a small macrocycle with bulky groups might also have the effect. Succinimide derivatives of *p-tert*-butyl-calix[4]arene **128** were chosen as a functionalized scaffold to which pyrrolidine derivatives were added. The acetamide fragments at the lower rim of the calixarene provided its ability to bind with alkali metal salts which in turn led to the rigid localization of the spatial structure of the dendrimer.

**Scheme 17:**
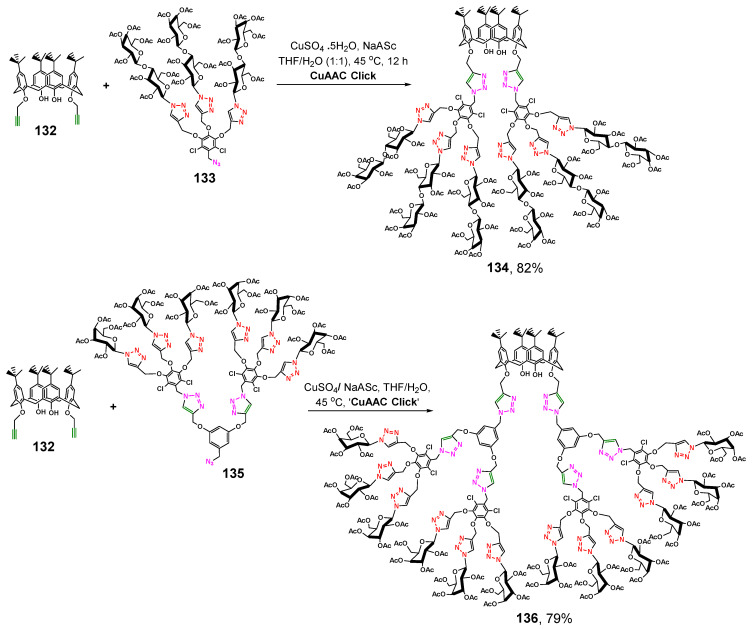
Synthesis of calix[4]arene-appended lactosylated G_1_ generation glycodendrimer **134** and galactosylated G_2_ generation glycodendrimer **136**.

The authors of [123] succeeded to achieve higher yields. The researchers chose bis-propargyloxy-*p-tert*-butyl-calix[4]arene as the starting reagent (Scheme 17). Using a regioselective Cu-catalyzed alkyne–azide cycloaddition reaction, they have obtained calix[4]arene-appended glycodendrimers of the first and second generations with lactose (**134**) and galactose (**136**) fragments, respectively. The presence of two functionalized propargyl groups only significantly reduced steric hindrances, so the yield of the target glycodendrimers was quite high.

**Scheme 18:**
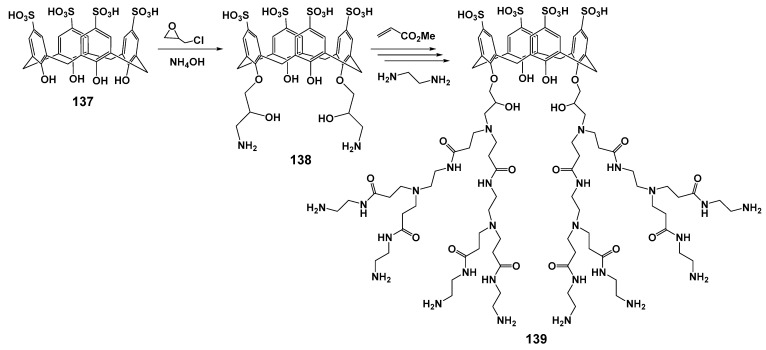
Grafting amine functional groups onto the surface of *p*-sulfonatocalix[4]arene, and preparation of calix-dendrimer **139** via performing the Michael addition and amidation reactions.

Using the divergent approach based on *p*-sulfonatocalix[4]arene **137**, the second generation of macrocyclic dendrimer with terminal amino groups **139** was obtained (Scheme 18) [124]. The resulting dendrimer was combined with Fe_3_O_4_ to obtain magnetic nanoparticles, which were the promising tools for the diagnosis and treatment of various diseases [125]. The resulting assembly was characterized by the optimal size of the associates formed in water (about 100 nm). The simultaneous presence of the hydrophobic macrocyclic cavity and anionic sulfonate groups, as well as cationic ammonium fragments in the dendrimer, opens up great prospects for binding a wide variety of substrates. The resulting dendrimer was proposed as a dual-drug carrier for the co-delivery of Doxorubicin and Methotrexate to MCF7 breast cancer cells with targeting and synergistic effects. The simultaneous presence of alkali and acidic fragments provided the possibility of drug releasing depending on pH. One important advantage of these magnetic nanoparticles was their low hemolytic activity and cytotoxicity towards the MCF7 cell line. Nanovehicle binding to human plasma proteins provided its high susceptibility to incorporation into cancer cells due to the presence of protein receptors at their surface.

### 2.4. Dendrimers with Addition Cyclophane Fragments in the Branches

**Figure 19:**
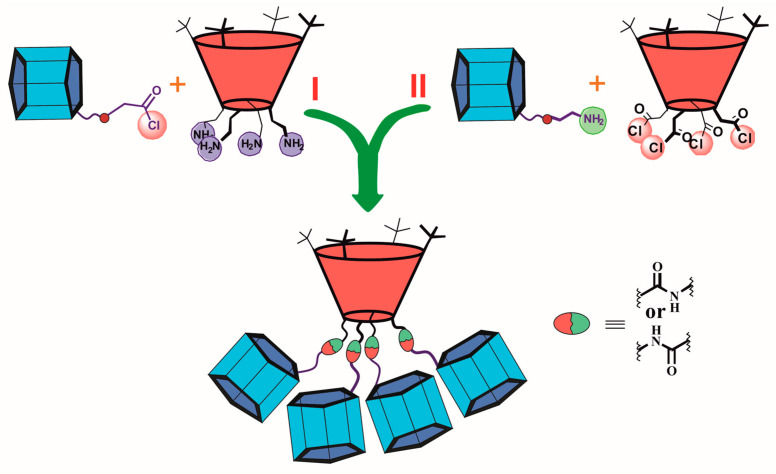
Two synthetic paths for hybrid multicyclophane preparation.

**Scheme 19:**
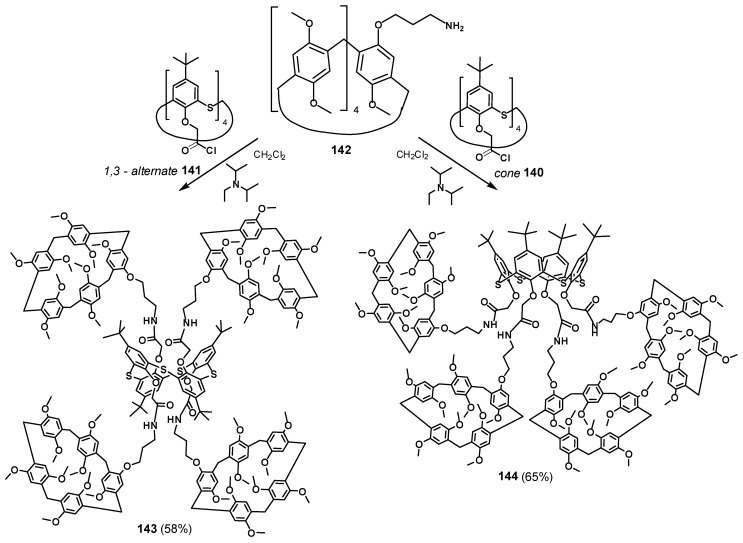
Synthesis of hybrid multicyclophanes **143** and **144**.

The dendrimers with macrocyclic compounds located at the periphery of the molecule are of great interest [126,127,128]. One exciting example is a hybrid multicyclophane molecule in which macrocycles are simultaneously presented both as a core (thiacalix[4]arene) and as the terminal fragments (pillar[5]arene) (Figure 19) [129]. The starting materials were acyl chlorides of *p-tert*-butylthiacalix[4]arene in the *cone* **140** and *1,3-alternate* **141** conformations, which were used to acylate the monoamino derivative of pillar[5]arene **142**. The target products **143** and **144** were obtained in satisfactory yields of 58 and 65%, respectively, (Scheme 19). The resulting compounds were capable of binding aniline and the salt of *p*-toluenesulfonic acid with aniline. The binding was performed with a pillararene fragment which could not bind *p*-toluenesulfonic acid in its pure form but could successfully bind the contact ion pair formed by *p*-toluenesulfonic acid and aniline forming an ensemble system. These properties provided the application of the obtained compounds for supramolecular assistance in aniline oxidative polymerization in aqueous *p*-toluenesulfonic acid solutions. In their presence, the yield of emeraldine increased from 50 to 90%. Simultaneously, its average molecular weight also increased. Hexa- and heptamer predominated among the polymerization products.

**Scheme 20:**
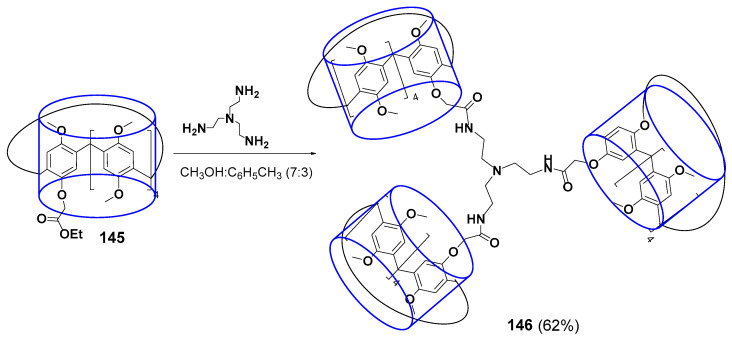
Synthesis of tris-pillar[5]arene **146**.

The multicyclophane with three terminal pillararene fragments **146** linked by amide linkers to a tris(2-aminoethyl)amine core was obtained in 62% yield (Scheme 20) [130]. The resulting compound could form the complex with *N*-phenyl-3-(phenylimino)-3H-phenothiazin-7-amine. Such complexation was accompanied by color change from pink for free phenothiazine to blue for the bound one. The phenothiazine dye color dependence on its environment was successfully used to create the colorimetric sensor for fluoride anion. The presence of fluoride in the solution led to the destruction of the multicyclophane complex with the dye followed by the formation of a new complex with the anion, whereas the color went back to pink upon phenothiazine releasing.

## 3. Supramolecular Cyclophane Dendrimers

**Scheme 21:**
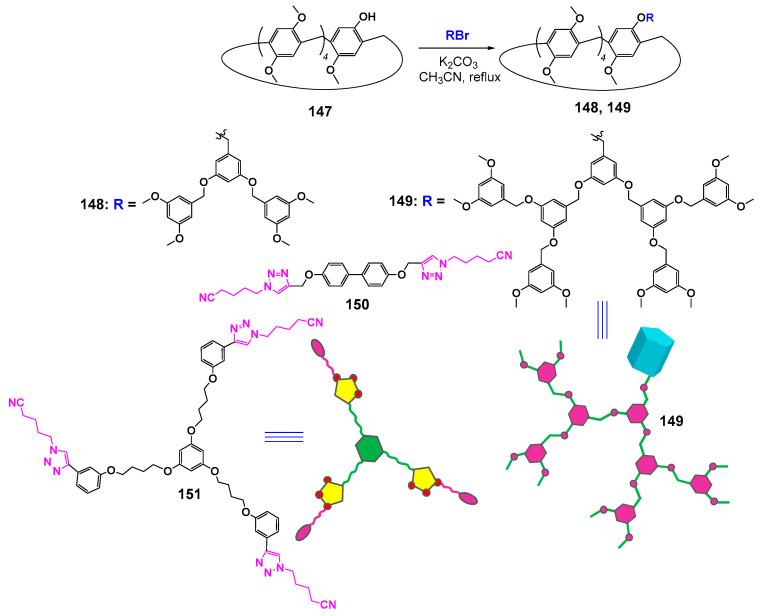
Synthesis of monosubstituted dendronized pillararenes and the structures of hosts and connectors.

**Figure 20:**
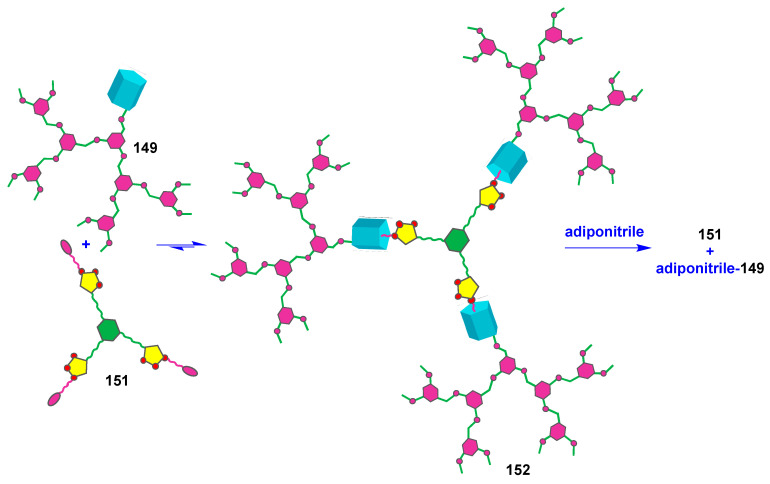
Schematic illustration of supramolecular trimeric dendrimer formation and its disassembly after adding a competitive guest.

Unfortunately, there were few investigations of supramolecular dendrimers based on cyclophanes. Thus, there are much more examples of macrocyclic dendrimers, whose formation occurred due to supramolecular interactions involving crown ethers and cyclodextrins [131,132,133]. At the same time, it is well known that pillararenes are able to form inclusion complexes with not quite bulky guests being fixed inside the cavity of the macrocycle. The formation of complexes occurs due to the negative electrostatic potential of pillararene. The polar guests can be embedded into the cavity if they have the charge distribution providing favorable electrostatic interactions with it [134]. The embedding of linear molecules with long alkyl fragments can be accompanied by the formation of pseudorotoxane structures [135]. This advantage of pillararenes was used by the authors of [136] to design supramolecular dendrimers based on pillar[5]arene **147**. For this purpose, the macrocycles monosubstituted with dendron fragments with one (**148**) or two branches (**149**) were obtained (Scheme 21). The resulting dendronized macrocycles were separated using column chromatography in good yields (60 and 76% in the case of dendrons with two and one branches, respectively). The authors achieved their further self-assembly into dimeric and trimeric dendrimers using di- and tritopic connectors (**150** and **151**) with a pentanenitrile fragment, which has a high binding constant with pillar[5]arene [137]. The process of supramolecular self-assembly was investigated by ^1^H NMR spectroscopy. The resulting supramolecular dendrimers were easily destroyed when a competitive guest was added. It was found, in the presence of adiponitrile, that the pentanenitrile fragment was displaced from the macrocyclic cavity of complex **152** with the following adiponitrile inserting in its place (Figure 20).

## 4. Conclusions

Thus, today cyclophane derivatives (pillar-, resorcinol-, (thia)calixarenes) are intensively studied as the components of dendrimers: both cores and terminal fragments. The combination of the hydrophobic platform with polar substituents allows the use of dendronized cyclophane derivatives for both polar and lipophilic compound binding. The simplicity of the functionalization of macrocyclic platforms and the possibility of the rigid fixation of substituents related to them in combination with the chiral properties of macrocycles allow the high-precision and inexpensive synthesis of compounds capable of selective binding to biosystems and influencing their properties. The possibility of introducing a large number of fragments at once including pharmaceutically active ones should be noted as giving unlimited prospects for new drug designs. Both covalent binding and supramolecular interactions are used for this purpose. The ability of thiacalixarene-based macrocyclic dendrimers to form supramolecular systems with DNA is successfully used to develop the electrochemical sensors for drugs and metabolite determination. Substrate binding is mainly provided by the terminal groups of cyclophane dendrimers or by the cavities formed by their branches with the fixation of the lipophilic guest molecule near the macrocyclic platform. The macrocyclic platform itself is directly involved in binding only during the formation of supramolecular dendrimers. Thus, currently, only the most common macrocyclic platforms (pillar[5]-, (thia)calix[4]-, calix[4]resorcinarenes) have been studied in detail as components of dendrimers. However, there is no data about the dendrimers based on their homologs. A promising direction of research is to expand the homologous set of macrocycles in the design of dendrimers, which will allow the involvement of additional centers (the macrocyclic platform itself) in substrate binding and expand the spectrum of detectable guest molecules. Thus, gaining new knowledge regarding cyclophane-based dendrimers can have great contribution to the improvement of a variety of technologies including the fields of alternative energy, electronics, catalysis, the development of new diagnostic tools and drugs. We hope that the further development of the topic presented will show broad opportunities for the synthesis of new materials with currently unavailable useful properties.

## Data Availability

Not applicable.
